# Analyzing the impact of heavy metal exposure on osteoarthritis and rheumatoid arthritis: an approach based on interpretable machine learning

**DOI:** 10.3389/fnut.2024.1422617

**Published:** 2024-07-19

**Authors:** Wenxuan Fan, Zhipeng Pi, Keyu Kong, Hua Qiao, Minghao Jin, Yongyun Chang, Jingwei Zhang, Huiwu Li

**Affiliations:** ^1^Shanghai Key Laboratory of Orthopaedic Implants, Department of Orthopaedic Surgery, Shanghai Ninth People’s Hospital, Shanghai Jiaotong University School of Medicine, Shanghai, China; ^2^School of International Pharmaceutical Business, China Pharmaceutical University, Nanjing, China

**Keywords:** machine learning, heavy metal exposure, NHANES, SHAP (SHapley Additive exPlanation), osteoarthritis and rheumatoid arthritis, environmental health

## Abstract

**Introduction:**

This investigation leverages advanced machine learning (ML) techniques to dissect the complex relationship between heavy metal exposure and its impacts on osteoarthritis (OA) and rheumatoid arthritis (RA). Utilizing a comprehensive dataset from the National Health and Nutrition Examination Survey (NHANES) spanning from 2003 to 2020, this study aims to elucidate the roles specific heavy metals play in the incidence and differentiation of OA and RA.

**Methods:**

Employing a phased ML strategy that encompasses a range of methodologies, including LASSO regression and SHapley Additive exPlanations (SHAP), our analytical framework integrates demographic, laboratory, and questionnaire data. Thirteen distinct ML models were applied across seven methodologies to enhance the predictability and interpretability of clinical outcomes. Each phase of model development was meticulously designed to progressively refine the algorithm’s performance.

**Results:**

The results reveal significant associations between certain heavy metals and an increased risk of arthritis. The phased ML approach enabled the precise identification of key predictors and their contributions to disease outcomes.

**Discussion:**

These findings offer new insights into potential pathways for early detection, prevention, and management strategies for arthritis associated with environmental exposures. By improving the interpretability of ML models, this research provides a potent tool for clinicians and researchers, facilitating a deeper understanding of the environmental determinants of arthritis.

## 1 Introduction

Arthritis is a debilitating disease characterized by joint inflammation, synovial swelling, stiffness, and potential cartilage damage. It has two common types: osteoarthritis (OA) and rheumatoid arthritis (RA) (1). Arthritis represents a significant global health concern. In the United States alone, 54 million people suffer from arthritis, and projections indicate that by 2040, nearly half of the population (49%) will be affected by this condition (2). Globally, the age-standardized point prevalence rates for osteoarthritis (3) and rheumatoid arthritis (4) in 2017 were 3.75 and 0.25%, respectively.

In recent years, the potential contributory role of heavy metals (HMs) in the exacerbation of arthritic conditions, particularly RA, has garnered research interest. A growing body of evidence suggests that heavy metals, such as cadmium (Cd) and lead (Pb), may exacerbate oxidative stress, leading to sustained inflammation (5)—a recognized factor in the pathogenesis of RA (6). Several epidemiological studies have corroborated a positive correlation between cadmium exposure and the incidence of RA (7, 8). However, the investigation into OA, another subtype of arthritis, and its association with heavy metal exposure remains underexplored in population-based epidemiological studies. While limited research has suggested possible links between heavy metal levels and OA, these are often confounded by factors such as aging and body mass index (BMI). Although limited studies have suggested a potential link between heavy metal levels and OA, these studies have either relied on traditional statistical methods or chosen a limited sample size (9). Additionally, approaches based on machine learning have not focused on the interpretability of the models (10). Moreover, considering OA as a critical differential diagnosis from RA, there is a conspicuous absence of studies examining the differential impact of heavy metals on these two arthritic subtypes, an area that warrants comprehensive investigation.

Research into the correlation between heavy metals and arthritis remains nascent, and existing studies predominantly rely on traditional statistical methods (11). These conventional approaches often necessitate extensive data requirements, incorporate numerous presumptions, and are subject to stringent application constraints, which restricts their capacity to derive insights from voluminous datasets. However, the dawn of the big data era, coupled with the swift advancement of computational technologies, has paved the way for the burgeoning application of machine learning (ML) techniques across various domains, including medical research. Machine learning, in particular, holds immense promise for enhancing disease prediction, diagnosis, and treatment paradigms. By processing and analyzing large datasets, ML algorithms are adept at uncovering intricate patterns and relationships that might otherwise remain undetected, thereby bolstering medical decision-making and advancing clinical practices.

In our investigation, we utilized a dataset from the National Health and Nutrition Examination Survey (NHANES) spanning from 2003 to 2020 to examine the link between heavy metal exposure and the prevalence of OA and RA. We employed seven distinct ML techniques, designed to discern the presence of arthritis attributable to heavy metal exposure, and assessed the predictive performance of each model. Furthermore, our study integrates the use of SHapley Additive exPlanations (SHAP)-based methodologies (12) to quantify the contribution of individual heavy metals to the accurate detection of arthritis. This approach not only elucidates the impact of heavy metals on the disease but also opens avenues for early intervention strategies.

Due to the unresolved issues highlighted above, this article investigates whether machine learning methods can effectively identify arthritis patients and distinguish between OA and RA. Additionally, it explores the correlation between heavy metal exposure and the incidence of arthritis, as well as determining which heavy metals play a crucial role in differentiating between OA and RA patients.

## 2 Methods

### 2.1 Participants of study

The dataset for our study was sourced from the NHANES, which employs a combination of questionnaire administration and physical examinations to collect comprehensive health data from the US population. The methodologies pertaining to these survey strategies have been extensively delineated in prior literature (13). For the purposes of our analysis, we included data from eight consecutive cycles of NHANES, covering the period from 2003 to 2020, to ensure a robust longitudinal perspective of the association between heavy metal exposure and arthritis.

Our study established the following inclusion criteria:

(1) Participants must be at least 20 years old.
(2) Participants must have taken part in the NHANES sub-study focusing on heavy metal analysis through blood and urine tests.
(3) Participants must have confirmable arthritis status information derived from the NHANES questionnaire data.

Conversely, the exclusion criteria were:

(1) Participants with missing data for more than two heavy metals out of a panel of 19.
(2) Participants with an arthritis status coded as 7 or 9 according to the NHANES questionnaire, indicative of an uncertain arthritis diagnosis, where 7 represents “Refused” and 9 represents “Do not know” in response to the question about arthritis status in the questionnaire.

After applying these criteria, the final cohort for analysis comprised 14,319 participants.

### 2.2 Data collection

#### 2.2.1 Demographic characteristics of the study participants

In our analysis, demographic and socioeconomic characteristics of the study participants were gleaned from the questionnaire data provided by NHANES. The collected characteristics encompass a broad spectrum, including gender, age, body mass index (BMI, expressed as kg/m^2^), racial/ethnic background (categorized broadly, including Hispanic and non-Hispanic classifications), educational attainment (categorized as college or above, high school or equivalent, and below high school), and the poverty income ratio (PIR), which was segmented into three groups for analytical purposes: below 1, between 1 to 4, and above 4. This diverse array of variables enables a comprehensive evaluation of the participants, facilitating a nuanced understanding of how demographic and socioeconomic factors may interact with heavy metal exposure to influence arthritis prevalence.

#### 2.2.2 Heavy metals

In our study, we conducted an analysis of 19 heavy metals present in the blood and urine samples of participants. The quantification of heavy metal concentrations was performed at the National Center for Environmental Health Laboratory, utilizing the highly precise method of inductively coupled plasma dynamic reaction cell mass spectrometry (ICP-DRCMS) (14). This technique is renowned for its sensitivity and accuracy in detecting trace levels of metals in biological samples. The application of such rigorous analytical steps ensures the reliability of the heavy metal exposure data, forming a crucial foundation for subsequent analyses examining the association between heavy metal exposure and the incidence of arthritis.

#### 2.2.3 Outcome ascertainment

In NHANES, the identification of arthritis among participants was based on self-reported data obtained from questionnaires. Initially, participants were asked to confirm whether they had been diagnosed with arthritis. Subsequently, for those who reported a diagnosis, the questionnaire data were used to ascertain the specific type of arthritis they had. This approach allowed for the differentiation between various forms of arthritis, such as RA, OA, and other subtypes, thereby facilitating a more nuanced analysis of the relationship between heavy metal exposure and specific arthritic conditions.

### 2.3 Pre-processing and extraction of ML features

The dataset underpinning our study comprised 25 features, with 21 being continuous variables and the remaining four categorized as categorical variables. To address missing values, we employed different strategies for each data type: median values were used to fill in missing continuous variables, while a nearest fill method was applied to categorical variables. This preparatory step ensured that the dataset was complete for subsequent analyses.

Our analysis leveraged the minimum absolute shrinkage and selection operator (LASSO) regression technique. LASSO (15) is particularly adept at handling datasets with numerous potential predictors, as it incorporates a penalty mechanism that reduces the regression coefficients of less significant variables towards zero. This feature of LASSO is instrumental in streamlining model complexity and mitigating the risk of overfitting, which is especially valuable when dealing with high-dimensional data. The intrinsic capability of LASSO to perform variable selection automatically is among its core strengths, enhancing both the model’s simplicity and its interpretive clarity while also potentially increasing prediction accuracy.

A crucial step in the application of LASSO regression in our study involved the standardization of variables (i.e., centralization and normalization), which is a prerequisite for the method to function optimally. Subsequently, the optimal penalty parameter, *λ*, was identified through a 5-fold cross-validation process. This approach to parameter tuning is critical for balancing the model’s complexity against its performance, ensuring that the selected variables are genuinely predictive of the study’s outcomes while minimizing the likelihood of incorporating spurious associations.

Through the application of LASSO regression analysis, our study has effectively pinpointed a suite of pivotal predictors. These key variables play an instrumental role in elucidating the underlying dynamics of our research phenomenon, thereby providing a solid foundation for both the formulation of subsequent experimental designs and the refinement of data analysis strategies. The process of identifying these predictors is critical, as it enables a focused investigation into the factors most relevant to the development of arthritis, ensuring that the research efforts are both efficient and directed towards areas of greatest potential impact.

The variables selected via the LASSO method were subsequently incorporated into a machine learning model. This step served a dual purpose: firstly, to validate the predictive power and relevance of these variables in the context of arthritis development, and secondly, to assess their contribution to the overall accuracy and performance of the predictive model. The integration of LASSO-selected variables into the machine learning framework not only confirms their significance but also enhances the model’s ability to make accurate predictions. This iterative process of variable selection and validation underscores the robustness of the methodology employed in our study, ensuring that the findings are both reliable and grounded in a rigorous analytical framework.

### 2.4 SMOTE sampling

In our study, we tackled the challenge of data imbalance—a common issue in medical datasets—using the synthetic minority oversampling technique (SMOTE) (16). This method is renowned for its effectiveness in addressing imbalances by artificially augmenting the size of the minority class through the generation of new instances. These instances are created via linear interpolation between existing minority class samples and their nearest neighbors, thus enriching the dataset without altering the majority class size. The application of SMOTE is not exclusive to our research; it has been extensively utilized across various medical studies, where it has consistently demonstrated its capability to enhance model performance by improving the recognition accuracy of underrepresented classes (17, 18).

The utility of SMOTE is particularly pronounced in our investigation into the development of arthritis, as it facilitates a more balanced distribution of classes within our dataset. By implementing SMOTE during the model’s training phase, we significantly boost the model’s ability to identify less prevalent categories. This improvement is pivotal for enhancing both the accuracy and the generalizability of our predictive model. It is important to note that SMOTE was exclusively applied to the training dataset to maintain the integrity and realism of the model evaluation process. The validation set was left in its original state, ensuring that the model’s performance could be accurately assessed on an untouched, representative sample of real-world data. This methodological choice underscores our commitment to ensuring the validity and reliability of our predictive model, enabling a faithful evaluation of its efficacy in novel contexts.

### 2.5 Machine learning strategy

**Figure 1:**
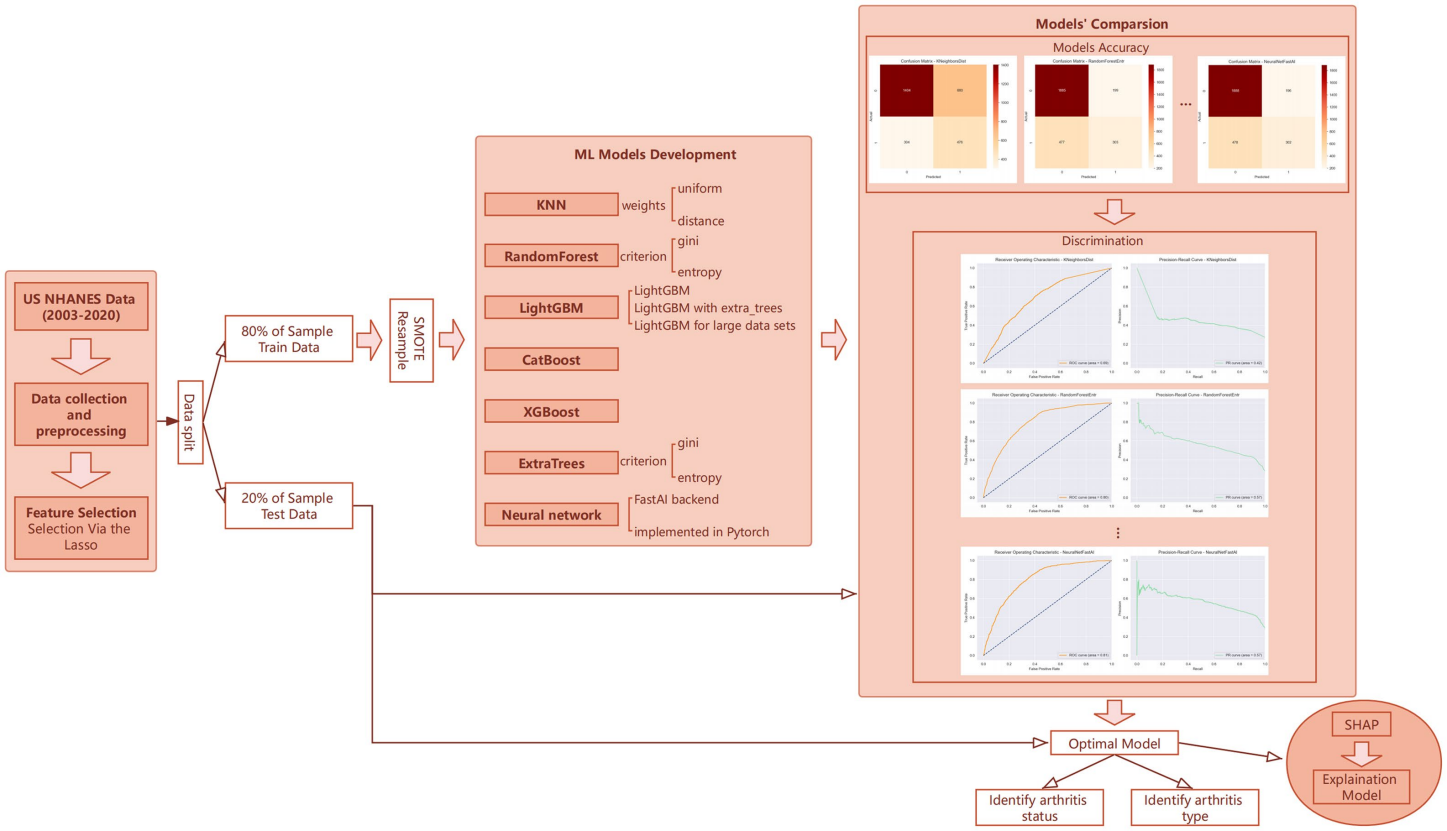
Overview plot.

In this study’s machine learning strategy, we employed the AutoGluon framework to construct and refine our predictive model through a phased approach. Initially, we developed 13 distinct machine learning models without leveraging advanced features such as auto-stacking, dynamic stacking, or hyperparameter optimization. As shown in Figure 1, these models included algorithms like K-Nearest Neighbors (KNN), RandomForest, ExtraTrees, LightGBM, CatBoost, XGBoost, and neural networks (19–24), each applied with various configurations and methodologies. This preliminary phase aimed to quickly generate a diverse set of models to explore the initial compatibility of our dataset with different machine learning algorithms.

Following the creation of these prototype models, we undertook a rigorous evaluation of their performance based on key metrics—accuracy, recall, and F1 score—using the validation set. Models exhibiting suboptimal performance were excluded from further consideration. For those models demonstrating promise, we then activated features such as auto-stacking, dynamic stacking, and engaged in hyperparameter optimization for retraining. Although this step entailed higher computational demands, the benefits in terms of enhanced prediction accuracy and model robustness were substantial, leading to the development of a suite of finely-tuned, high-quality machine learning models.

Ultimately, the model that showcased superior performance on the test set was selected as our predictive model. This process not only confirmed the model’s exceptional predictive accuracy but also validated the efficacy of our phased machine learning strategy. Through this methodical and iterative approach, we were able to systematically identify and optimize the most effective machine learning solution for predicting arthritis development, underscoring the strategic advantage of employing a phased methodology in machine learning projects.

### 2.6 Statistical analysis

In this study, we meticulously detailed the demographic characteristics of the participants. Continuous variables were summarized using the median and interquartile range, while categorical variables were presented as counts and percentages. To discern differences in characteristics based on arthritis status, we employed the Wilcoxon two-sample test for continuous variables and the chi-square test for categorical variables. Heavy metal exposure levels across eight NHANES data release cycles were reported using geometric mean and geometric standard deviation, with the Mann–Kendall test applied to assess trend significance over time.

The performance of our machine learning model was evaluated using several metrics, including the area under the curve (AUC) as described by Pruessner et al. (25), accuracy score, average precision score (APS), sensitivity/recall, and the F1 score. Given the imbalance in our dataset—marked by significant discrepancies in the prevalence of positive and negative samples—the average precision (AP) metric was deemed more appropriate for evaluating the binary classification model’s performance regarding arthritis status. The AP provides a nuanced measure of sensitivity and discriminative ability in unbalanced settings, hence its prioritization in our analysis. For the arthritis classification, which involves multiple classes, the F1 macro score was selected as the primary evaluation criterion due to its capacity to offer a balanced view of precision and recall rates across an unbalanced dataset.

Our analysis was conducted using Python version 3.9.18 and R version 4.3.2, provided by The R Foundation for Statistical Computing. Results achieving a *p*-value less than 0.05 were considered statistically significant. This methodological framework, as depicted in Figure 1, outlines our comprehensive approach to understanding the impact of heavy metal exposure on arthritis development, supported by robust statistical and machine learning analyses.

### 2.7 SHAP interpretation

SHAP (SHapley Additive exPlanations) (12) is an advanced model interpretation tool that draws upon the principles of cooperative game theory, specifically utilizing the concept of Shapley values, to elucidate the decision-making processes of machine learning models. The foundational theory behind SHAP posits that each feature within a model can be seen as a “player” in a cooperative game, where the “payout” or impact of each player is determined by their contribution to the predictive accuracy of the model. SHAP operates on the principle of “fair distribution,” akin to “more work, more gain,” ensuring that the contribution of each feature to the model’s prediction is accurately quantified and allocated.

This method demystifies the predictive judgments of complex models by detailing the exact contribution of each feature to the final prediction, thereby rendering the model’s decision-making process transparent. One of the hallmark characteristics of SHAP is its versatility and compatibility; it can be applied *post hoc* to virtually any machine learning model regardless of its underlying architecture. This is achieved by decomposing the model’s output into a linear sum of the individual contributions of all features. By converting the predicted value into the aggregated sum of these attributions, SHAP facilitates a deeper understanding of the model’s behavior, highlighting which features are most influential in driving predictions and providing insights into the dynamics underlying the model’s predictions.

## 3 Results

### 3.1 Population characteristics of the study participants

Table 1 in our study encapsulates the demographic and health-related characteristics of participants enrolled in the U.S. National Health and Nutrition Examination Survey (NHANES) between 2003 and 2020, focusing specifically on individuals with and without arthritis. The analysis included a cohort of 14,319 participants, with a gender distribution where 49% were male. The average age across the cohort was 49.0 years, with an interquartile range from 34.0 to 63.0 years. Within this population, a significant number, 3,900 participants, were identified as suffering from arthritis.

**Table 1:** Characteristics of the study participants from 2003–2020 in US NHANES.

Characteristic	Arthritis status				Arthritis type				
	Overall, *N* = 14,319 (100%)^a^	Without, *N* = 10,419 (73%)^a^	With, *N* = 3,900 (27%)^a^	*p*-value^b^	Overall, *N* = 1,536 (100%)^a^	Osteoarthritis, *N* = 904 (59%)^a^	Other or unknown, *N* = 248 (16%)^a^	Rheumatoid arthritis, *N* = 384 (25%)^a^	*p*-value^b^
**Age (years)**	49 (34, 64)	43 (31, 58)	63 (53, 73)	**<0.001**	63 (54, 72)	65 (56, 73)	59 (48, 69)	62 (53, 71)	**<0.001**
**Sex**				**<0.001**					**<0.001**
*Male*	7,020 (49%)	5,357 (51%)	1,663 (43%)		628 (41%)	331 (37%)	115 (46%)	182 (47%)	
*Female*	7,299 (51%)	5,062 (49%)	2,237 (57%)		908 (59%)	573 (63%)	133 (54%)	202 (53%)	
**BMI**	28.0 (24.3, 32.5)	27.4 (24.0, 31.8)	29.4 (25.6, 34.4)	**<0.001**	29.5 (26.0, 34.6)	29.9 (26.1, 34.6)	29.2 (25.6, 34.6)	29.3 (26.1, 34.6)	0.8
**Race**				**<0.001**					**<0.001**
*Mexican American*	2,283 (16%)	1,868 (18%)	415 (11%)		133 (8.7%)	70 (7.7%)	13 (5.2%)	50 (13%)	
*Other Hispanic*	1,304 (9.1%)	985 (9.5%)	319 (8.2%)		136 (8.9%)	72 (8.0%)	26 (10%)	38 (9.9%)	
*Non-Hispanic White*	6,087 (43%)	4,036 (39%)	2,051 (53%)		765 (50%)	514 (57%)	115 (46%)	136 (35%)	
*Non-Hispanic Black*	3,099 (22%)	2,250 (22%)	849 (22%)		346 (23%)	158 (17%)	63 (25%)	125 (33%)	
*Other*	1,546 (11%)	1,280 (12%)	266 (6.8%)		156 (10%)	90 (10.0%)	31 (13%)	35 (9.1%)	
**Education Level**				**<0.001**					**0.011**
*Less than high school*	3,591 (25%)	2,474 (24%)	1,117 (29%)		338 (22%)	185 (20%)	49 (20%)	104 (27%)	
*High school*	3,358 (23%)	2,358 (23%)	1,000 (26%)		367 (24%)	205 (23%)	73 (29%)	89 (23%)	
*More than high school*	7,361 (51%)	5,581 (54%)	1,780 (46%)		830 (54%)	514 (57%)	126 (51%)	190 (50%)	
**PIR Level**				**<0.001**					**<0.001**
*Low*	2,680 (21%)	1,913 (20%)	767 (22%)		301 (22%)	153 (19%)	53 (24%)	95 (28%)	
*Medium*	7,085 (54%)	5,118 (54%)	1,967 (56%)		746 (54%)	443 (54%)	120 (54%)	183 (53%)	
*High*	3,284 (25%)	2,475 (26%)	809 (23%)		345 (25%)	231 (28%)	49 (22%)	65 (19%)	

a Median (IQR); *n* (unweighted) (%).

b Wilcoxon rank-sum test for complex survey samples; chi-squared test with Rao & Scott’s second-order correction.The bold values indicate significant differences in demographic and health characteristics by arthritis status.

The comparative analysis between participants with arthritis and those without highlighted several notable demographic and socioeconomic distinctions. Specifically, individuals diagnosed with arthritis were predominantly female, tended to be older, and were more likely to identify as non-Hispanic white. Additionally, this group was characterized by a moderate household income. These differences between the two groups were statistically significant, with all comparisons yielding a *p*-value of less than 0.05.

### 3.2 Data of heavy metal exposure

Table 2 in our study presents a detailed analysis of the concentrations of various heavy metals detected in urine or blood samples across each data release cycle from the U.S. National Health and Nutrition Examination Survey (NHANES). The heavy metals examined include total arsenic, arsenite, arsenic acid, dimethylarsinic acid, monomethylarsonic acid, barium, cadmium, lead, antimony, and tungsten, in addition to the specific analysis of cadmium and lead levels in blood samples.

**Table 2:** Means and standard deviations of heavy metals by each cycle of US NHANES (2003–2020).

Variable	Overall, *N* = 14,319 (100%)^a^	Cycles of US NHANES								*p* for trend^b^
		2003–2004, *N* = 1,496 (10%)^a^	2005–2006, *N* = 1,453 (10%)^a^	2007–2008, *N* = 1711 (12%)^a^	2009–2010, *N* = 1946 (14%)^a^	2011–2012, *N* = 1,627 (11%)^a^	2013–2014, *N* = 1735 (12%)^a^	2015–2016, *N* = 1707 (12%)^a^	2017–2020, *N* = 2,644 (18%)^a^	
**In urine**										
Total arsenic (μg/L)	21.60 (58.40)	19.97 (52.35)	24.43 (70.39)	18.82 (53.08)	23.89 (61.45)	20.75 (53.06)	0.00 (0.00)	0.00 (0.00)	0.00 (0.00)	**0.001**
Arsenous acid (μg/L)	0.65 (1.51)	0.85 (0.39)	0.90 (0.63)	0.97 (3.76)	0.92 (0.73)	0.56 (1.68)	0.50 (0.48)	0.38 (0.45)	0.32 (0.46)	**<0.001**
Arsenic acid (μg/L)	0.68 (1.33)	0.75 (0.29)	0.76 (0.44)	0.80 (3.56)	0.75 (0.65)	0.67 (1.06)	0.58 (0.26)	0.58 (0.15)	0.59 (0.20)	**<0.001**
Arsenobetaine (μg/L)	10.77 (46.71)	9.68 (41.69)	13.60 (56.85)	8.71 (38.56)	11.60 (41.61)	12.08 (45.41)	9.36 (39.83)	10.04 (32.77)	11.18 (61.72)	**<0.001**
Arsenocholine (μg/L)	0.31 (1.15)	0.42 (0.21)	0.45 (0.30)	0.44 (0.31)	0.55 (2.83)	0.25 (0.43)	0.15 (0.53)	0.17 (0.47)	0.17 (0.77)	**<0.001**
Dimethylarsonic acid (μg/L)	5.52 (7.86)	5.46 (6.20)	5.83 (9.37)	5.52 (8.71)	6.02 (9.08)	6.40 (8.50)	4.94 (5.97)	5.11 (6.51)	5.14 (7.66)	**<0.001**
Monomethylarsonic acid (μg/L)	0.81 (1.88)	0.97 (0.80)	0.98 (1.18)	0.98 (1.43)	1.07 (4.26)	0.93 (1.89)	0.62 (0.75)	0.54 (0.54)	0.53 (0.63)	**<0.001**
Barium (μg/L)	1.91 (3.25)	2.13 (3.49)	2.23 (3.29)	2.16 (4.10)	2.11 (3.37)	1.75 (2.97)	1.69 (2.99)	1.75 (2.77)	1.64 (2.92)	**<0.001**
Cadmium (μg/L)	0.39 (0.47)	0.45 (0.51)	0.41 (0.43)	0.43 (0.49)	0.40 (0.47)	0.38 (0.51)	0.32 (0.42)	0.34 (0.38)	0.37 (0.48)	**<0.001**
Cobalt (μg/L)	0.54 (1.56)	0.53 (3.39)	0.60 (1.60)	0.49 (0.63)	0.53 (1.20)	0.47 (1.00)	0.55 (1.17)	0.59 (1.14)	0.54 (1.25)	**<0.001**
Cesium (μg/L)	5.32 (6.73)	6.52 (17.61)	5.78 (4.31)	5.43 (4.34)	5.01 (3.22)	4.85 (3.35)	4.88 (3.24)	5.07 (4.28)	5.29 (3.60)	**<0.001**
Lead (μg/L)	0.69 (1.17)	0.96 (0.93)	0.95 (1.23)	0.85 (1.62)	0.81 (1.65)	0.64 (1.23)	0.51 (0.87)	0.51 (0.75)	0.48 (0.66)	**<0.001**
Antimony (μg/L)	0.09 (0.22)	0.10 (0.10)	0.11 (0.16)	0.09 (0.17)	0.08 (0.15)	0.07 (0.12)	0.07 (0.16)	0.09 (0.47)	0.08 (0.21)	**<0.001**
Thallium (μg/L)	0.19 (0.15)	0.18 (0.13)	0.19 (0.13)	0.18 (0.15)	0.18 (0.13)	0.19 (0.14)	0.18 (0.13)	0.20 (0.23)	0.20 (0.15)	**<0.001**
Tungsten (μg/L)	0.12 (0.37)	0.11 (0.21)	0.14 (0.32)	0.16 (0.32)	0.12 (0.24)	0.14 (0.84)	0.10 (0.21)	0.11 (0.24)	0.10 (0.22)	**<0.001**
Molybdenum (μg/L)	53.40 (52.23)	54.73 (57.24)	59.23 (52.38)	60.47 (56.87)	57.16 (55.02)	52.95 (51.72)	47.75 (53.08)	49.96 (44.10)	48.30 (47.26)	**<0.001**
**In blood**										
Cadmium (μg/L)	0.51 (0.58)	0.55 (0.59)	0.52 (0.57)	0.55 (0.62)	0.53 (0.57)	0.53 (0.59)	0.48 (0.54)	0.48 (0.54)	0.50 (0.58)	**<0.001**
Lead (μg/dL)	1.58 (1.56)	2.08 (1.56)	1.89 (1.54)	1.88 (1.81)	1.69 (1.71)	1.50 (1.79)	1.34 (1.45)	1.30 (1.30)	1.23 (1.12)	**<0.001**
Total Mercury (μg/L)	1.55 (2.54)	1.47 (1.90)	1.55 (2.01)	1.49 (2.18)	1.68 (2.54)	1.73 (3.03)	1.57 (2.69)	1.57 (2.59)	1.43 (2.84)	**<0.001**

a Mean (SD).

b Wilcoxon rank-sum test for complex survey samples.The bold values represent significant trends or variations in heavy metal exposure levels across different NHANES cycles.

The analysis reveals a significant trend in the concentration levels of these heavy metals over the data release cycles, with a *p*-value of less than 0.05 indicating statistical significance. This suggests that there has been a consistent and noteworthy variation in the exposure levels to these metals among the U.S. population during the study period.

### 3.3 Training and testing of machine learning models

In the initial phase of our study, we focused on developing machine learning models capable of identifying the presence of arthritis. To refine the feature set for our machine learning (ML) models, we employed the LASSO regression technique for feature selection. This approach enabled us to identify 21 variables that exhibited non-zero coefficients after LASSO’s regularization process, indicating their significance in predicting arthritis.

**Figure 2:**
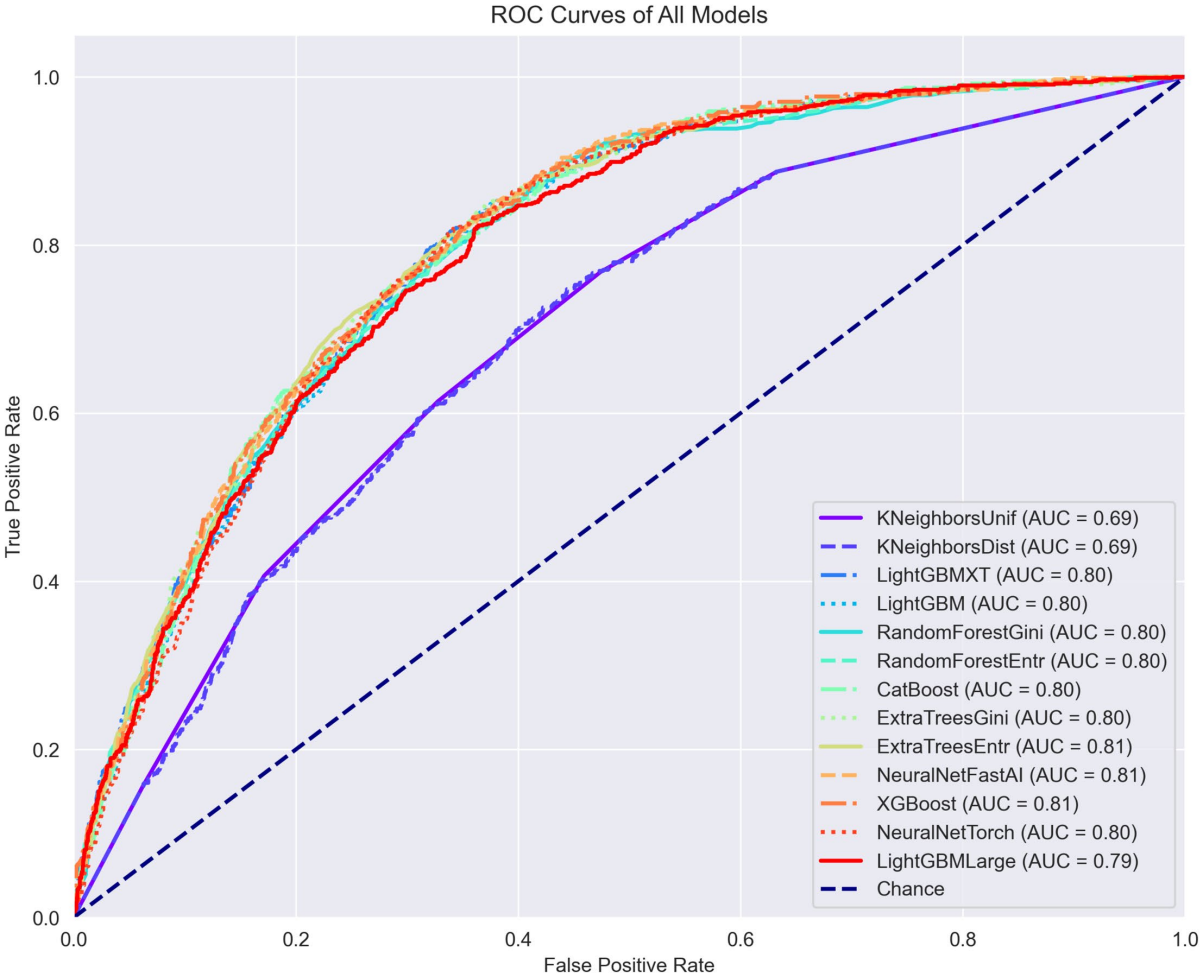
ROC curves, PR curves, and confusion matrices for each model in the binary classification task of diagnosing arthritis.

The training outcomes of this first stage are documented in figures within the Supplementary material. Figure 2 illustrates the receiver operating characteristic (ROC) curves of the various ML models trained, plotted together for comparative analysis. It is important to note that certain models, such as K-nearest neighbors (KNN), demonstrated notably inferior performance in this specific task. As a result, models performing suboptimally were subsequently excluded from further analysis in favor of those that could be optimized with more effective settings.

**Figure 3:**
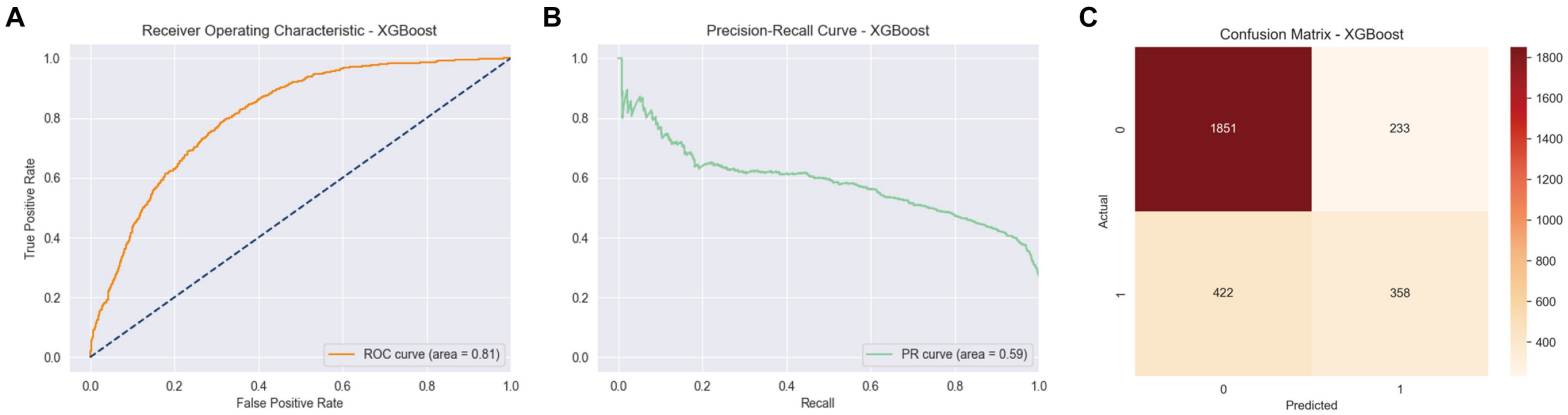
ROC curve, PR curve, confusion matrix of stage II XGBoost in the binary classification task of identifying arthritis. **(A)** ROC Curve for XGBoost model, showing the True Positive Rate against the False Positive Rate with an AUC of 0.81. **(B)** PR Curve for XGBoost model. **(C)** Confusion Matrix for XGBoost model, displaying the actual vs. predicted values.

Among the various models evaluated, XGBoost emerged as particularly effective in predicting arthritis, showcasing an area under the curve (AUC) of 0.81, an accuracy of 0.77, an average precision score (APS) of 0.59, a precision of 0.61, a recall of 0.50, and an F1 score of 0.54. These results, depicted in Figure 3, underscore the superior performance of XGBoost in this context.

Following the successful determination of arthritic status among participants, our study’s next objective was to differentiate between osteoarthritis (OA) and rheumatoid arthritis (RA) and to explore the specific influence of heavy metal exposure on these forms of arthritis. Leveraging the arthritis patient data initially selected, we proceeded with a similar two-stage machine learning model training approach. This process began with the extraction of relevant features from the dataset comprising identified arthritis patients, aiming to refine and select the most effective models through a rigorous two-stage training regimen. This strategy was designed to isolate the models that exhibited the strongest performance on the test set, with the ultimate goal of utilizing the optimized model to investigate the relationship between heavy metal exposure and the different types of arthritis. Such an approach not only aids in the precise categorization of arthritis types but also in understanding their potential associations with environmental factors.

**Figure 4:**
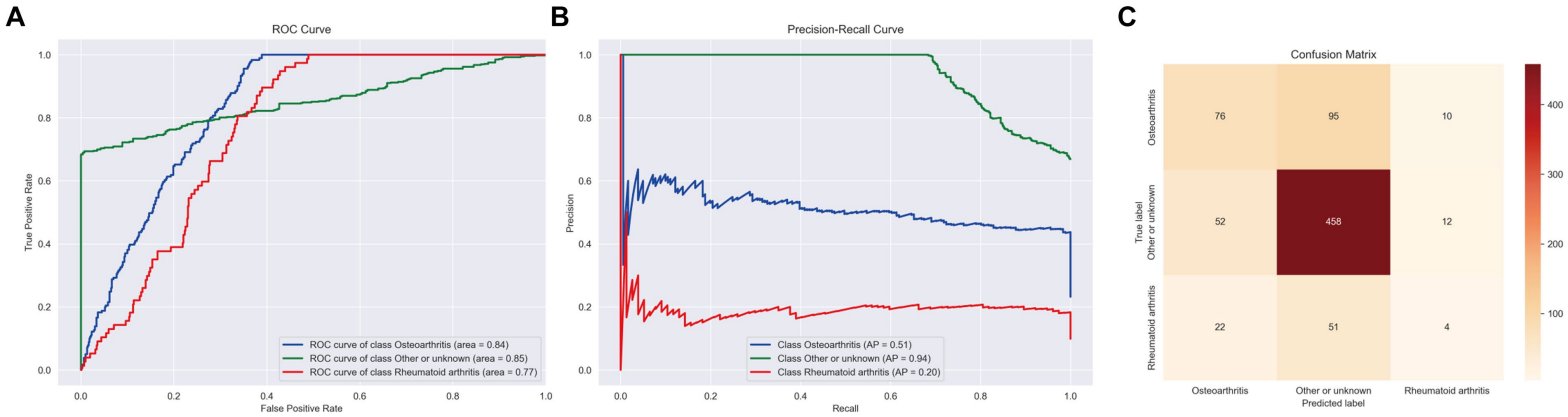
ROC curve, PR curve, confusion matrix of stage II LightGBM in the multi-classification task of identifying arthritis species. **(A)** ROC Curves for different classes. **(B)** PR Curves for different classes. **(C)** Confusion Matrix displaying the actual vs. predicted values for the different classes.

In the final analysis, the LightGBM_Large model emerged as particularly effective for this task, achieving a macro area under the curve (AUC) of 0.76, an accuracy of 0.70, a balanced accuracy of 0.53, and a macro F1 score of 0.85. These outcomes are detailed in Figure 4.

### 3.4 Feature importance visualization

**Figure 5:**
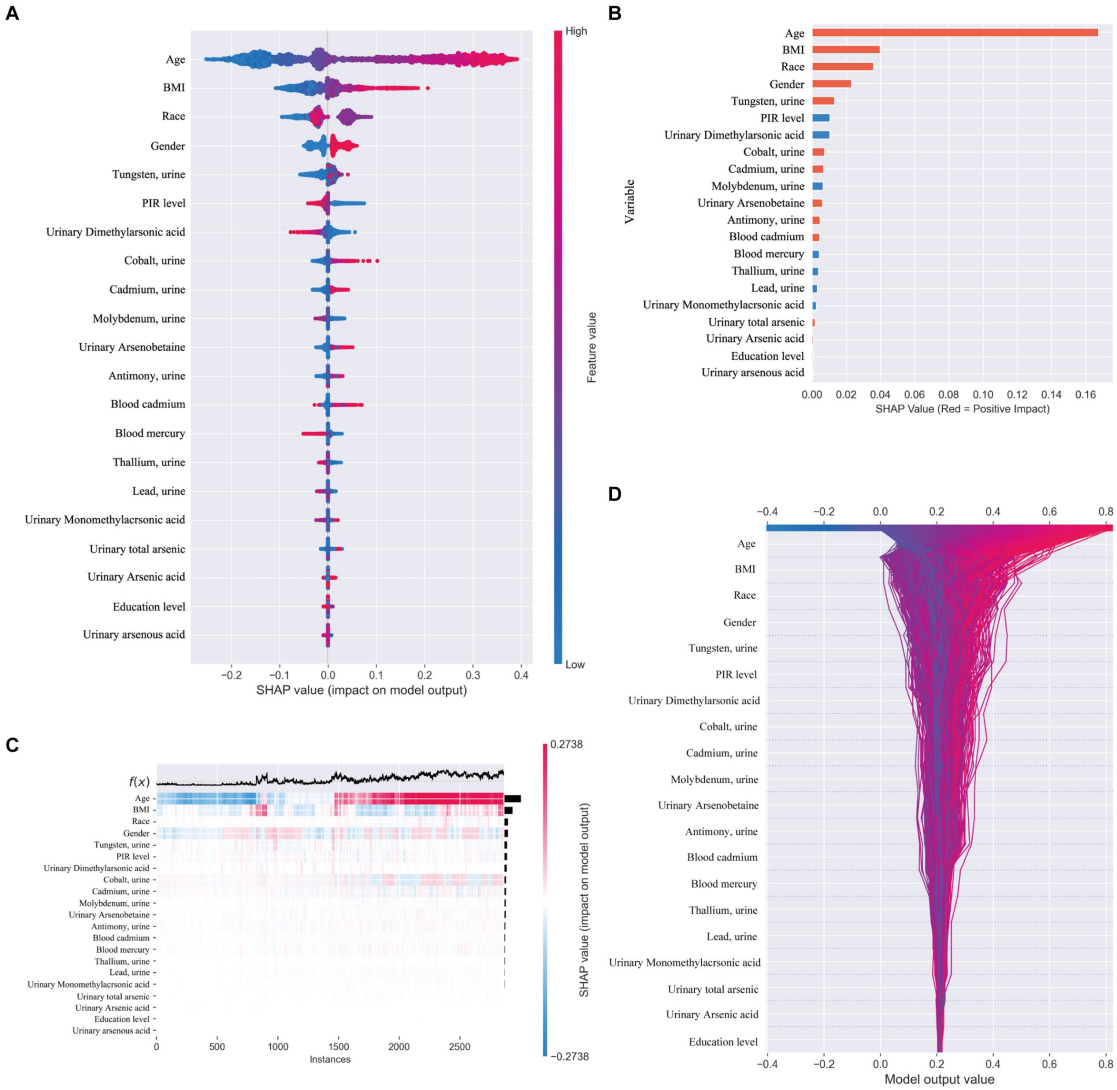
**(A)** SHAP plot. Top-to-bottom features are sorted by the average of the absolute shell values, that is, the vertical position shows the importance of the features. Each point in the figure represents the SHAP value for each sample, the color represents the feature value (the red high, the blue low), and the horizontal position shows whether the effect of this value leads to higher or lower predictions. **(B)** The features shown in red indicate positive contributions to the model, such as urinary tungsten (0.013), cobalt (0.007), cadmium (0.007), antimony (0.005), total arsenic (0.002), and cadmium in blood (0.005). The features shown in blue indicate a negative contribution to the model, such as molybdenum (−0.007) in urine, thallium (−0.004), lead (−0.003), and mercury in blood (−0.004). **(C)** SHAP heatmap. **(D)** SHAP decision diagram.

In our study, we utilized SHAP (SHapley Additive exPlanations) to illuminate the impact of each variable within the XGBoost model on the status of arthritis in the test dataset. SHAP plots, including a decision map and a heatmap, visualize the model’s decision-making process and the distribution of SHAP values for each feature, respectively, as depicted in Figure 5. The analysis of SHAP values revealed that certain heavy metals significantly contribute to the model’s predictions. Specifically, tungsten (0.013) in urine and other metals such as cobalt (0.007), cadmium (0.007), antimony (0.005), total arsenic (0.002), and blood cadmium (0.005) showed positive contributions, indicating their association with an increased likelihood of arthritis diagnosis. Conversely, molybdenum in urine (−0.007), thallium (−0.004), lead (−0.003), and mercury (−0.004) in blood demonstrated negative contributions, suggesting their inverse relationship with arthritis risk.

Beyond heavy metal exposure, demographic factors like gender, age, and ethnicity (non-Hispanic whites) were also identified as significant, with these groups showing a higher association with arthritis prevalence. This underscores the multifaceted nature of arthritis risk, encompassing both environmental and demographic influences.

**Figure 6:**
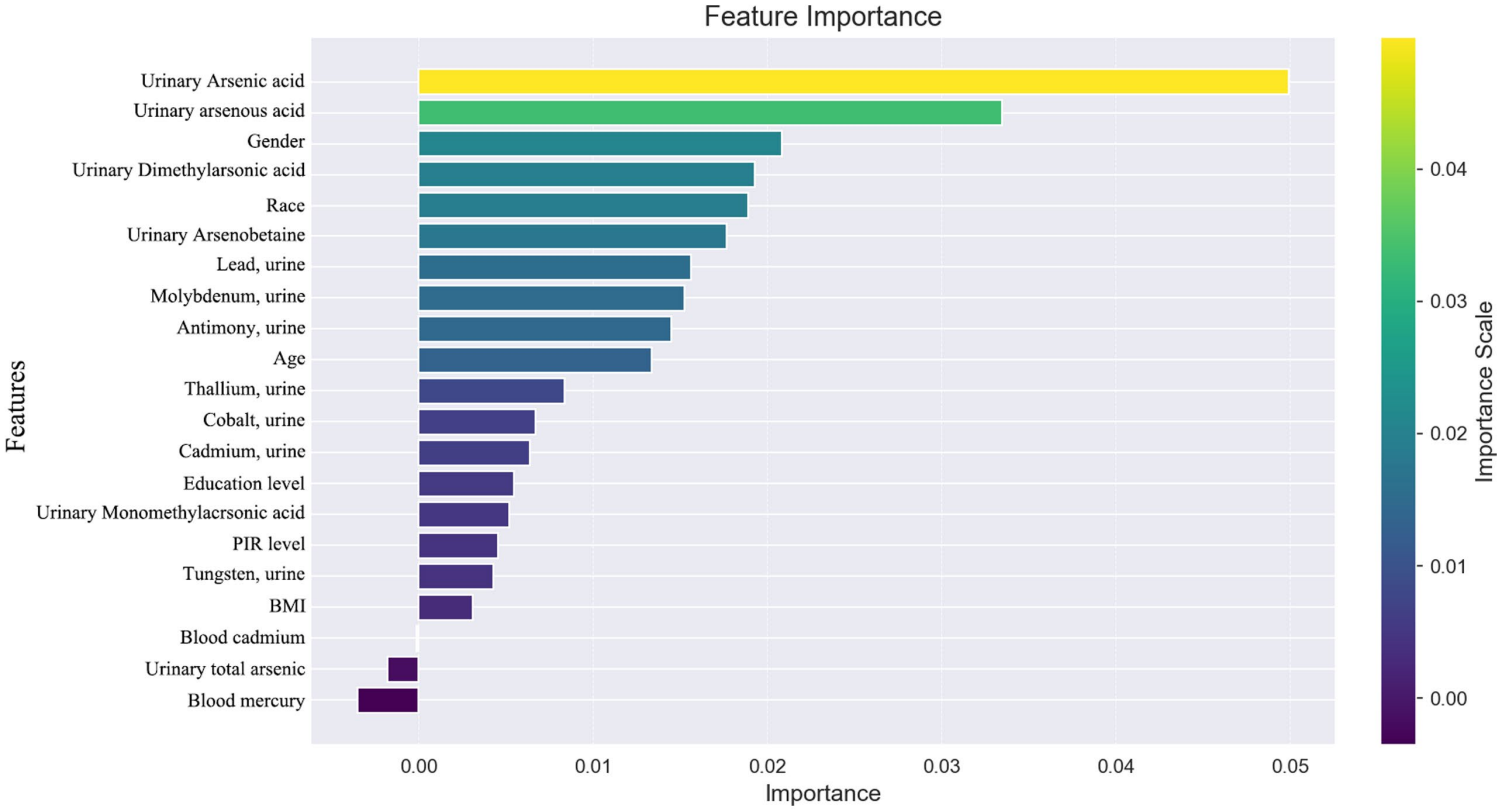
Feature importance based on permutation shuffling.

**Figure 7:**
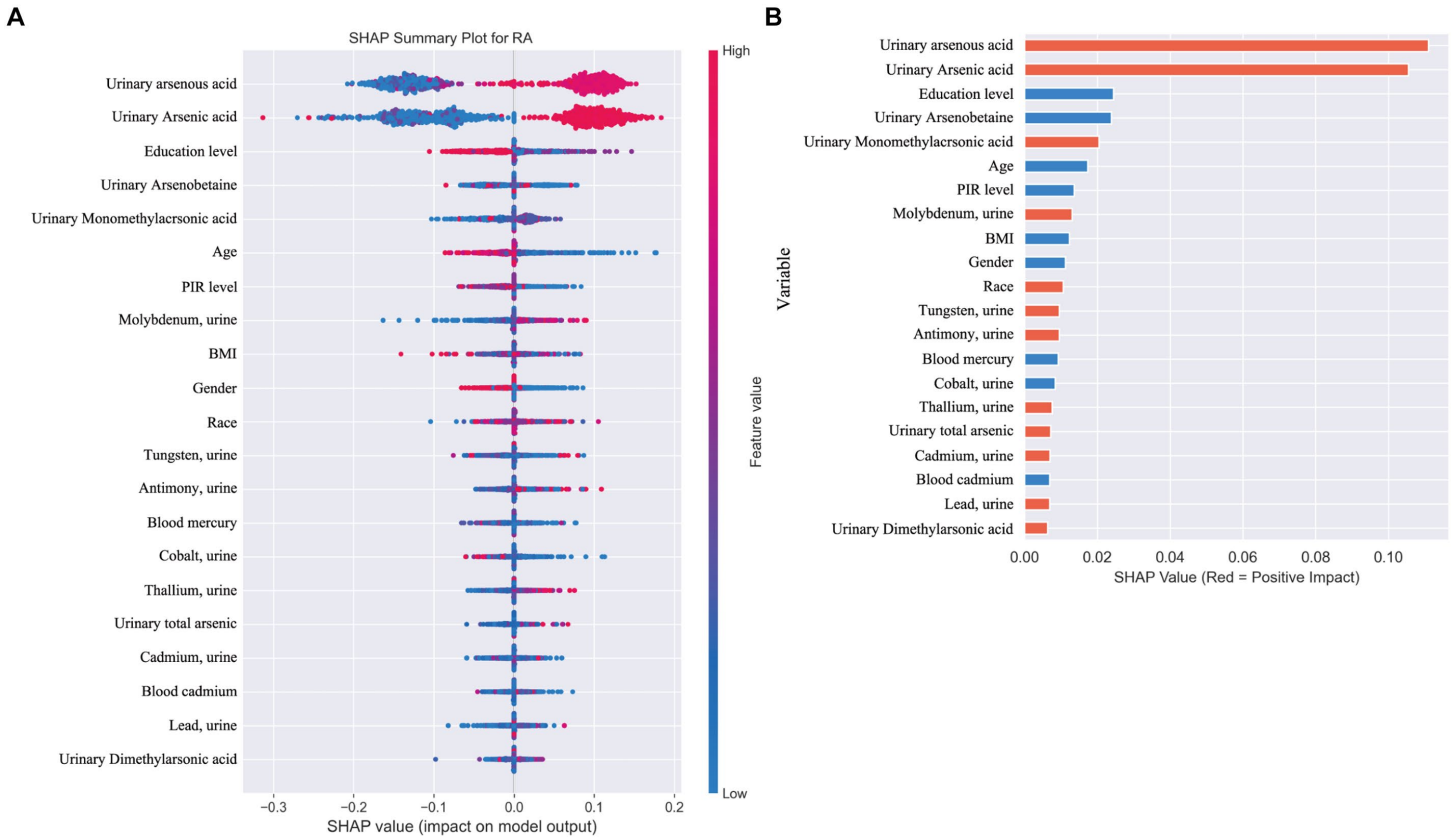
**(A)** SHAP plot. This figure illustrates the application of a machine learning model in a binary classification task for identifying the presence of rheumatoid arthritis (RA). **(B)** Positive (red) SHAP values indicate features that contribute to predicting RA presence, including arsenic metabolites (0.02), molybdenum (0.013), tungsten (0.009), antimony (0.009), and thallium (0.007). Negative (blue) SHAP values, such as for mercury (−0.009) and cobalt (−0.008), suggest these features are more associated with other types of arthritis or osteoarthritis (OA).

When distinguishing between specific types of arthritis (OA, RA, or unspecified arthritis), the SHAP analysis for the multi-classification task did not yield as straightforward an interpretation as the binary classification model. Hence, we resorted to using feature importance based on permutation shuffling to ascertain the significance of heavy metal exposure in identifying OA and RA, with findings presented in Figure 6. Additionally, the development of RA was examined as a binary classification issue, exploring how the machine learning model discerns RA presence through SHAP values, detailed in Figure 7.

## 4 Discussion

In this investigation, we employed LASSO regression to efficiently delineate key variables and adopted explainable machine learning methodologies linked to heavy metal exposure. These techniques were implemented to ascertain the presence of arthritis within the NHANES database spanning 2003 to 2020. In our meticulous analysis of heavy metal exposure’s ramifications on OA and RA, the XGBoost and LightGBM algorithms demonstrated exceptional prowess in managing the complexities of the dataset, with XGBoost attaining an AUC of 0.81 and a precision rate of 77%, whereas LightGBM achieved an AUC of 0.76 and an accuracy of 70%. Both models were augmented by SHAP values, affording profound insights into how heavy metals modulate the risk trajectories for OA and RA, thereby enhancing the interpretability of our models and informing subsequent research trajectories.

ML, an intricate branch of artificial intelligence, leverages sophisticated mathematical algorithms to parse and categorize patterns across disparate datasets, thereby bolstering decision-making processes. Despite its efficacy, the opaque reasoning mechanisms of ML algorithms and the complexity inherent in their interpretability pose significant challenges to their practical application in medical decision-making (26). Our machine learning strategy boasted several distinctive features. Primarily, it circumvented the need for new data acquisition, instead exploiting demographic, laboratory, and questionnaire data from the NHANES, applying multi-source data to our machine learning models. Furthermore, our models underwent training and evaluation on an extensive dataset, with a particular emphasis on the blood levels of heavy metals in individual participants. Given that the annual average levels of heavy metal exposure among the study participants were not incorporated into the training data, the decreasing trends in metal content did not compromise model stability. Additionally, our phased machine learning strategy, which was congruent with NHANES’s questionnaire collection methodology, initially determined whether participants suffered from arthritis, subsequently classifying the type of arthritis. The AUC of XGBoost and LightGBM in these phased tasks stood at 0.81 and 0.76, respectively, indicative of robust model stability. In addition, our study implemented machine learning models configured with 13 distinct parameter settings, based on 7 different methodologies, to evaluate the efficacy of machine learning in our research context. The application of SHAP values to the XGBoost and LightGBM models was intended to more effectively illustrate the decision-making processes of the machine learning models. Positive SHAP values indicated a heightened risk of OA and RA during the 18-year survey period of the United States NHANES, whereas negative values suggested a reduced risk.

SHAP outcomes aligned with prior research, pinpointing exposures most closely linked with arthritis development as tungsten (0.013), cobalt (0.007), cadmium (0.007), antimony (0.005), and arsenic (0.002), significant as potential risk factors. In individuals diagnosed with arthritis, the presence of arsenic, lead, molybdenum, antimony, thallium, cobalt, cadmium, and tungsten significantly differentiated between OA and RA based on levels of heavy metal exposure.

Presently, studies on how tungsten, cobalt, and antimony catalyze the mechanisms that induce OA and RA remain sparse. Our findings offer valuable perspectives for future investigations into the impact of these heavy metals on the pathogenesis of arthritis. Cadmium (Cd), a known environmental contaminant causing renal damage and bone demineralization, has been demonstrated to promote the expression of enzymes linked to the breakdown of the extracellular matrix in joint cartilage and diminish the presence of glycosaminoglycans and proteoglycans through the generation of reactive oxygen species (27–29). Recent studies have highlighted arsenic as a contributory factor in the development of arthritis (29). In a mouse model integrated with surgery-induced joint instability (30), arsenic’s presence markedly intensified cartilage degradation, consistent with our findings. Prior studies correlated mercury with the onset of osteoarthritis, noting elevated mercury levels in the anterior cruciate ligaments of women under 65 with degenerative spinal conditions (31). The presence of mercury in bones may correlate with body mass index, anatomical differences, and sex (32). Our classification of the arthritis-affected populace reaffirmed the linkage between mercury and OA. Investigations by Grech (33) have shown that molybdenum could counteract iron-deficiency anemia by increasing enzyme quantities, revitalizing enzyme activity, and impeding inflammatory pathways, suggesting molybdenum’s role as a protective agent against arthritis, a finding corroborated by our analysis. Although certain studies have explored the relationship between blood lead levels and the prevalence and severity of knee osteoarthritis (34), our research did not substantiate this connection, likely due to the cross-sectional study design’s inherent limitations.

Moving forward, continuous monitoring and elucidation of selected features will yield invaluable insights for experts, enabling them to formulate well-founded conclusions instead of merely accepting the algorithm’s outputs. Furthermore, we intend to focus on validating the performance of the model by broadening the database and augmenting the interpretability of the interface between clinicians and the machine learning model.

This study has its limitations, including the absence of longitudinal follow-up for the same cohort and the current inability to access other datasets of similar scale for validation. We plan to address these issues in future research. Additionally, the inherent biases of cross-sectional studies, potential information bias from self-reported arthritis diagnoses, and biases resulting from imputing missing data are also limitations. Moreover, disparities in feature importance between permutation shuffling and SHAP, with the former concentrating on global explanations and the latter on individual prediction contributions, may impede replicability due to the complexity entailed in model interpretation.

## 5 Conclusion

Our study effectively utilized phased machine learning strategies to investigate the link between heavy metal exposure and arthritis prevalence among NHANES participants from 2003 to 2020. Employing SHAP enhanced our understanding of the predictive outcomes of these models, providing deep insights into the factors contributing to arthritis. This approach combines advanced analytics with improved interpretability, overcoming the typical “black box” issue in machine learning and enabling a more detailed exploration of the relationship between environmental exposures and health outcomes.

## Data availability statement

The original contributions presented in the study are included in the article/Supplementary material, further inquiries can be directed to the corresponding authors.

## Ethics statement

The studies involving humans were approved by the data analyzed in this study are from the National Health and Nutrition Examination Survey (NHANES), which received ethical approval from the Research Ethics Review Board (ERB) at the National Center for Health Statistics (NCHS), which is part of the Centers for Disease Control and Prevention (CDC). The studies were conducted in accordance with the local legislation and institutional requirements. Written informed consent for participation was not required from the participants or the participants’ legal guardians/next of kin in accordance with the national legislation and institutional requirements.

## Author contributions

WF: Data curation, Formal analysis, Methodology, Validation, Visualization, Writing – original draft. ZP: Writing – review & editing, Data curation, Validation. KK: Writing – review & editing. HQ: Writing – review & editing. MJ: Writing – review & editing. YC: Writing – review & editing. JZ: Writing – review & editing. HL: Supervision, Writing – review & editing.

## References

[1] Tang C-H. Research of pathogenesis and novel therapeutics in arthritis. Int J Mol Sci. (2019) 20:1646. doi: 10.3390/ijms20071646, PMID: 30987068 PMC6479975

[2] Barbour KE. Vital signs: prevalence of doctor-diagnosed arthritis and arthritis-attributable activity limitation—United States, 2013–2015. MMWR Morb Mortal Wkly Rep. (2017) 66:246–53. doi: 10.15585/mmwr.mm6609e1 28278145 PMC5687192

[3] Safiri S Kolahi AA Hoy D Smith E Bettampadi D Mansournia MA. Global, regional and national burden of rheumatoid arthritis 1990–2017: a systematic analysis of the Global Burden of Disease Study 2017. Ann Rheum Dis. (2019) 78:1463–71. doi: 10.1136/annrheumdis-2019-215920, PMID: 31511227

[4] Safiri S Kolahi A-A Smith E Hill C Bettampadi D Mansournia MA. Global, regional and national burden of osteoarthritis 1990–2017: a systematic analysis of the Global Burden of Disease Study 2017. Ann Rheum Dis. (2020) 79:819–28. doi: 10.1136/annrheumdis-2019-216515, PMID: 32398285

[5] Paithankar JG Saini S Dwivedi S Sharma A Chowdhuri DK. Heavy metal associated health hazards: an interplay of oxidative stress and signal transduction. Chemosphere. (2021) 262:128350. doi: 10.1016/j.chemosphere.2020.128350, PMID: 33182141

[6] Smallwood MJ Nissim A Knight AR Whiteman M Haigh R Winyard PG. Oxidative stress in autoimmune rheumatic diseases. Free Radic Biol Med. (2018) 125:3–14. doi: 10.1016/j.freeradbiomed.2018.05.086 29859343

[7] Chen L Sun Q Peng S Tan T Mei G Chen H. Associations of blood and urinary heavy metals with rheumatoid arthritis risk among adults in NHANES, 1999–2018. Chemosphere. (2022) 289:133147. doi: 10.1016/j.chemosphere.2021.133147, PMID: 34864016

[8] Joo SH Lee J Hutchinson D Song YW. Prevalence of rheumatoid arthritis in relation to serum cadmium concentrations: cross-sectional study using Korean National Health and Nutrition Examination Survey (KNHANES) data. BMJ Open. (2019) 9:e023233. doi: 10.1136/bmjopen-2018-023233, PMID: 30610019 PMC6326419

[9] Guan T Wu Z Xu C Su G. The association of trace elements with arthritis in US adults: NHANES 2013–2016. J Trace Elem Med Biol. (2023) 76:127122. doi: 10.1016/j.jtemb.2022.127122, PMID: 36525916

[10] Xia F Li Q Luo X Wu J. Identification for heavy metals exposure on osteoarthritis among aging people and machine learning for prediction: a study based on NHANES 2011–2020. Front Public Health. (2022) 10:906774. doi: 10.3389/fpubh.2022.906774, PMID: 35979456 PMC9376265

[11] Fang L Zhao H Chen Y Ma Y Shanshan X Shenqian X. The combined effect of heavy metals and polycyclic aromatic hydrocarbons on arthritis, especially osteoarthritis, in the U.S. adult population. Chemosphere. (2023) 316:137870. doi: 10.1016/j.chemosphere.2023.137870 36642150

[12] Lundberg S M Lee S-I. (2017). A unified approach to interpreting model predictions. Advances in Neural Information Processing Systems. Curran Associates, Inc.. Available at: https://proceedings.neurips.cc/paper/2017/hash/8a20a8621978632d76c43dfd28b67767-Abstract.html

[13] National Center for Health Statistics. (2023). Surveys and data collection systems. Available at: https://www.cdc.gov/nchs/surveys.htm

[14] National Center for Health Statistics. (2024). NHANES 2013–2014 laboratory methods. Available at: https://wwwn.cdc.gov/nchs/nhanes/ContinuousNhanes/LabMethods.aspx?BeginYear=2013. (Accessed March 30, 2024)

[15] Tibshirani R. Regression shrinkage and selection via the LASSO. J R Stat Soc B. (1996) 58:267–88. doi: 10.1111/j.2517-6161.1996.tb02080.x

[16] Chawla NV Bowyer KW Hall LO Kegelmeyer WP. SMOTE: synthetic minority over-sampling technique. J Artif Intell Res. (2002) 16:321–57. doi: 10.1613/jair.953

[17] López V Fernández A García S Palade V Herrera F. An insight into classification with imbalanced data: empirical results and current trends on using data intrinsic characteristics. Inf Sci. (2013) 250:113–41. doi: 10.1016/j.ins.2013.07.007

[18] Sung S-F Hung L-C Ya-Han H. Developing a stroke alert trigger for clinical decision support at emergency triage using machine learning. Int J Med Inform. (2021) 152:104505. doi: 10.1016/j.ijmedinf.2021.104505, PMID: 34030088

[19] Breiman L. Random forests. Mach Learn. (2001) 45:5–32. doi: 10.1023/A:1010933404324

[20] Geurts P Ernst D Wehenkel L. Extremely randomized trees. Mach Learn. (2006) 63:3–42. doi: 10.1007/s10994-006-6226-1

[21] Ke G Meng Q Finley T Wang T Chen W Ma W. (2017). LightGBM: a highly efficient gradient boosting decision tree. Advances in Neural Information Processing Systems. Curran Associates, Inc.. Available at: https://papers.nips.cc/paper_files/paper/2017/hash/6449f44a102fde848669bdd9eb6b76fa-Abstract.html

[22] Wikipedia. (2024). K-nearest neighbors algorithm. Wikipedia. Available at: https://en.wikipedia.org/w/index.php?title=K-nearest_neighbors_algorithm&oldid=1212348037

[23] Prokhorenkova L Gusev G Vorobev A Dorogush AV Gulin A. (2019). CatBoost: unbiased boosting with categorical features. *arXiv*. Available at: 10.48550/arXiv.1706.09516. [Epub ahead of preprint]

[24] Chen T Guestrin C. (2016). XGBoost: a scalable tree boosting system. Proceedings of the 22nd ACM SIGKDD International Conference on Knowledge Discovery and Data Mining. 785–794

[25] Pruessner JC Kirschbaum C Meinlschmid G Hellhammer DH. Two formulas for computation of the area under the curve represent measures of total hormone concentration versus time-dependent change. Psychoneuroendocrinology. (2003) 28:916–31. doi: 10.1016/S0306-4530(02)00108-7, PMID: 12892658

[26] de Souza A Alvaro AP Stubbs DA Baan CC Boer K. Cherry on top or real need? A review of explainable machine learning in kidney transplantation. Transplantation. (2024). doi: 10.1097/TP.0000000000005063, PMID: 38773859

[27] Frangos T Maret W. Zinc and cadmium in the aetiology and pathogenesis of osteoarthritis and rheumatoid arthritis. Nutrients. (2021) 13:53. doi: 10.3390/nu13010053, PMID: 33375344 PMC7824316

[28] Liu H Liu M Qiao L Yang Z He Y Bao M. Association of blood cadmium levels and all-cause mortality among adults with rheumatoid arthritis: the NHANES cohort study. J Trace Elem Med Biol. (2024) 83:127406. doi: 10.1016/j.jtemb.2024.127406, PMID: 38308912

[29] Skalny AV Aschner M Zhang F Guo X Djordevic AB Sotnikova TI. Molecular mechanisms of environmental pollutant-induced cartilage damage: from developmental disorders to osteoarthritis. Arch Toxicol. (2024). doi: 10.1007/s00204-024-03772-9, PMID: 38758407

[30] Suminda GG Dinesh YM Ha MW Ghosh M Lee D-S Son Y-O. *In vitro* and *in vivo* investigations on arsenic-induced cartilage degeneration in osteoarthritis. J Hazard Mater. (2024) 461:132570. doi: 10.1016/j.jhazmat.2023.132570, PMID: 37742380

[31] Zioła-Frankowska A Dąbrowski M Kubaszewski Ł Rogala P Kowalski A Frankowski M. An analysis of factors affecting the mercury content in the human femoral bone. Environ Sci Pollut Res. (2017) 24:547–57. doi: 10.1007/s11356-016-7784-9, PMID: 27734315 PMC5219028

[32] Pamphlett R Jew SK. Mercury is taken up selectively by cells involved in joint, bone, and connective tissue disorders. Front Med. (2019) 6:168. doi: 10.3389/fmed.2019.00168, PMID: 31380381 PMC6659129

[33] Grech BJ. Mechanistic insights into the treatment of iron-deficiency anemia and arthritis in humans with dietary molybdenum. Eur J Clin Nutr. (2021) 75:1170–5. doi: 10.1038/s41430-020-00845-7, PMID: 33514867

[34] Nelson AE Chaudhary S Kraus VB Fang F Chen J-C Schwartz TA. Whole blood lead levels are associated with biomarkers of joint tissue metabolism in African American and white men and women: the Johnston County Osteoarthritis Project. Environ Res. (2011) 111:1208–14. doi: 10.1016/j.envres.2011.08.002, PMID: 21839992 PMC3210895

