# Supplementary figures and images for: Analyzing the impact of heavy metal exposure on osteoarthritis and rheumatoid arthritis: an approach based on interpretable machine learning
Source: Front Nutr. 2024 Jul 19;11:1422617. doi: 10.3389/fnut.2024.1422617 (PMC11294220; doi:10.3389/fnut.2024.1422617)

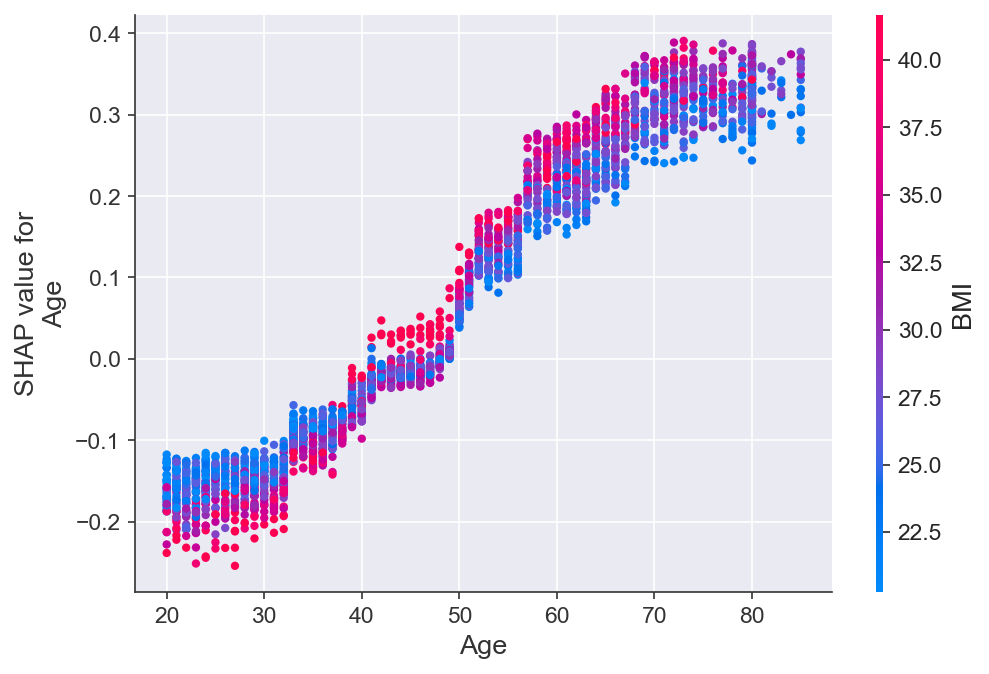

Supplement: Supplementary file 1 [file Data_Sheet_1.zip › dependence/Age.png]

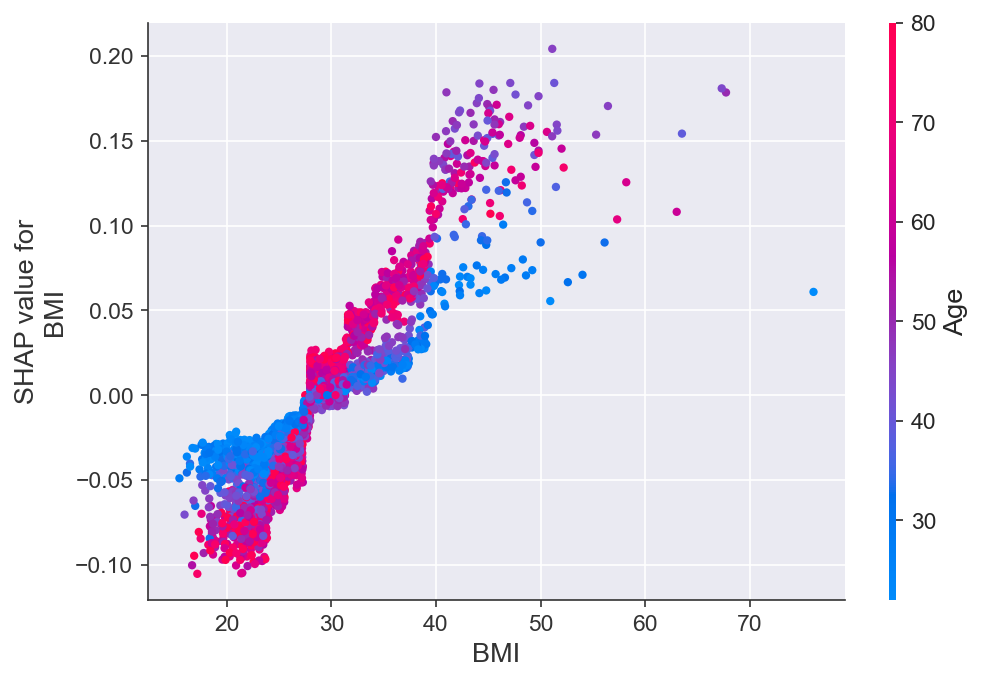

Supplement: Supplementary file 1 [file Data_Sheet_1.zip › dependence/BMI.png]

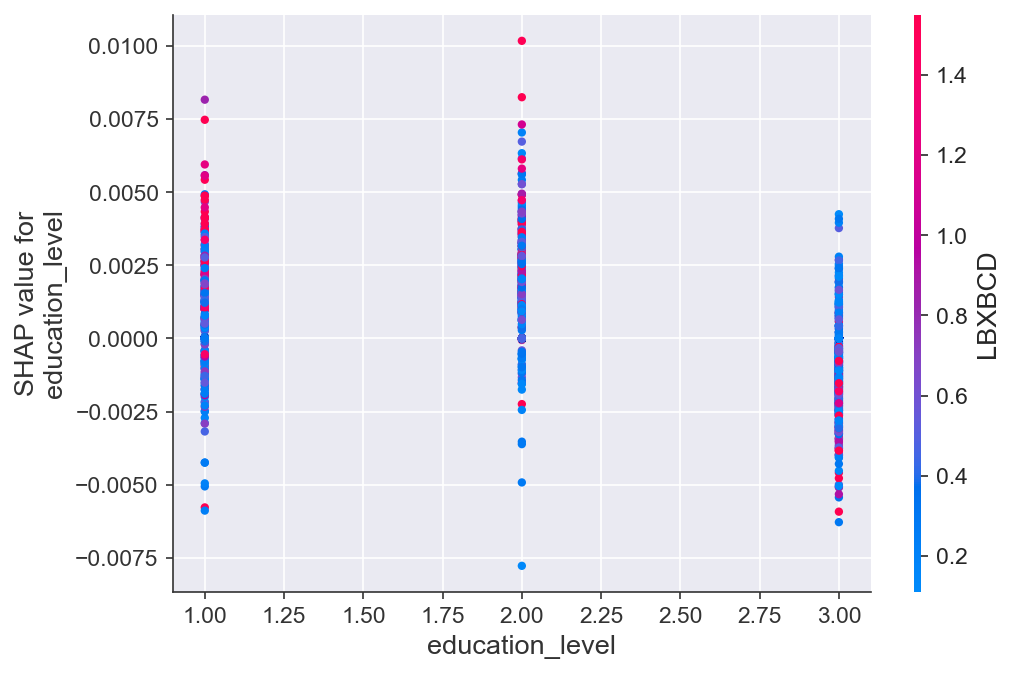

Supplement: Supplementary file 1 [file Data_Sheet_1.zip › dependence/education_level.png]

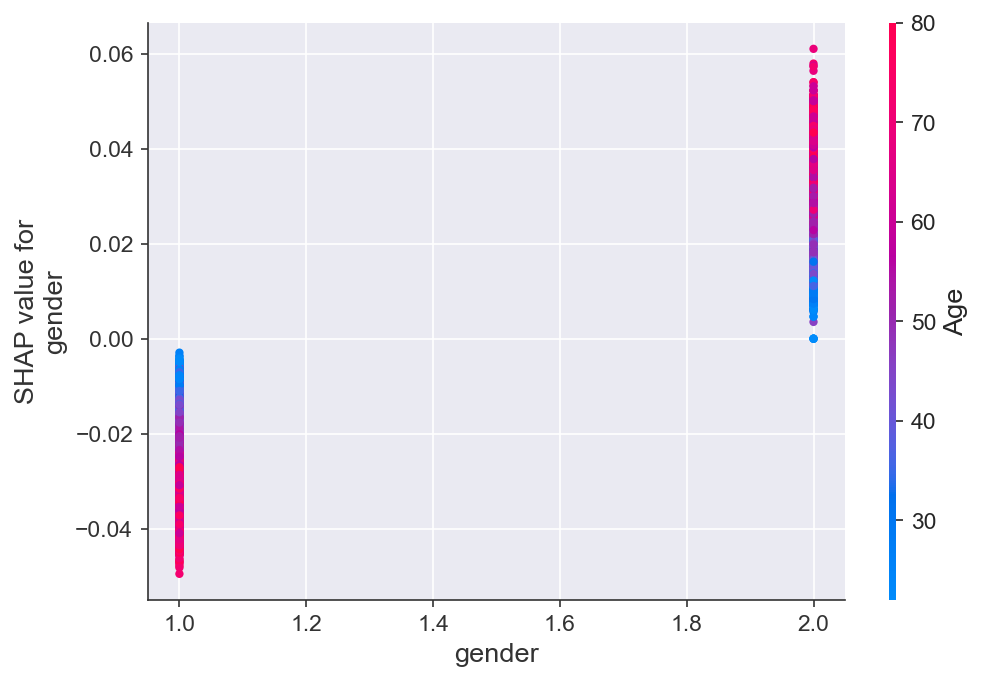

Supplement: Supplementary file 1 [file Data_Sheet_1.zip › dependence/gender.png]

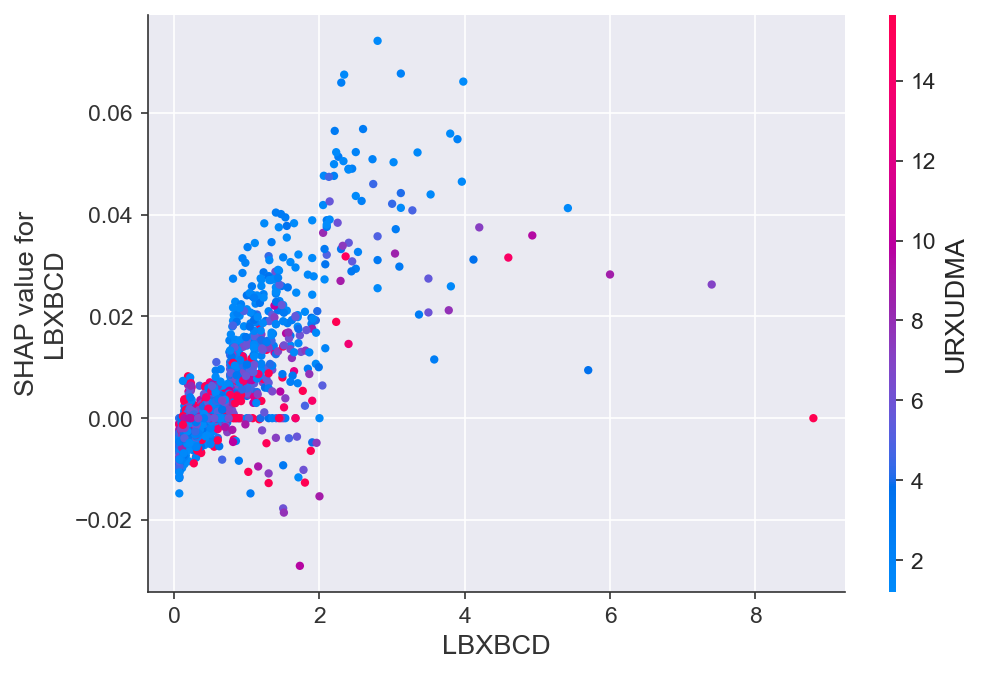

Supplement: Supplementary file 1 [file Data_Sheet_1.zip › dependence/LBXBCD.png]

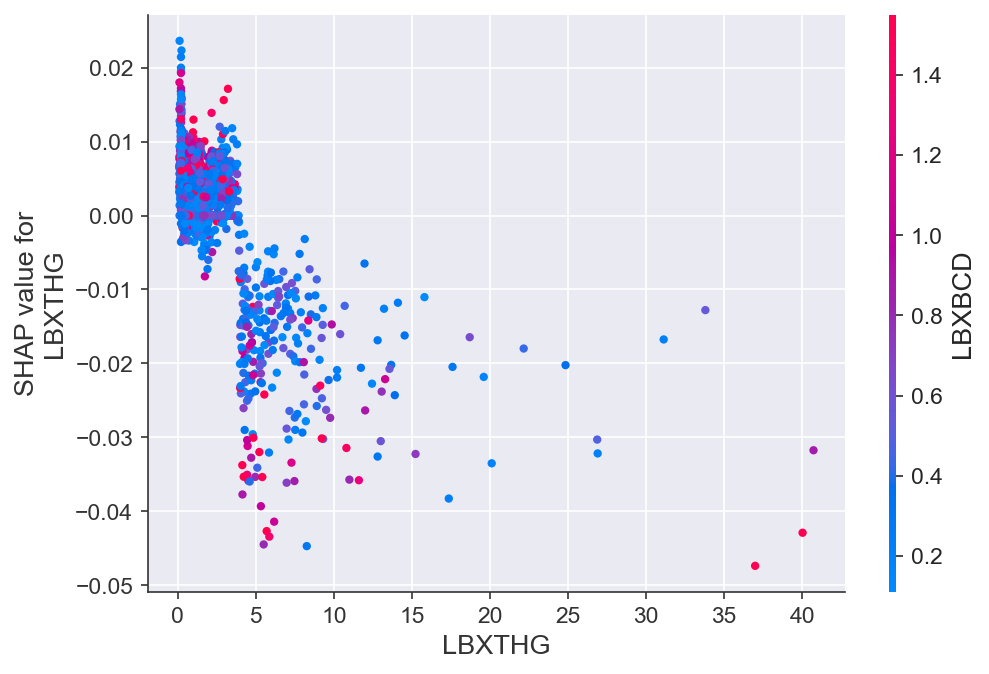

Supplement: Supplementary file 1 [file Data_Sheet_1.zip › dependence/LBXTHG.png]

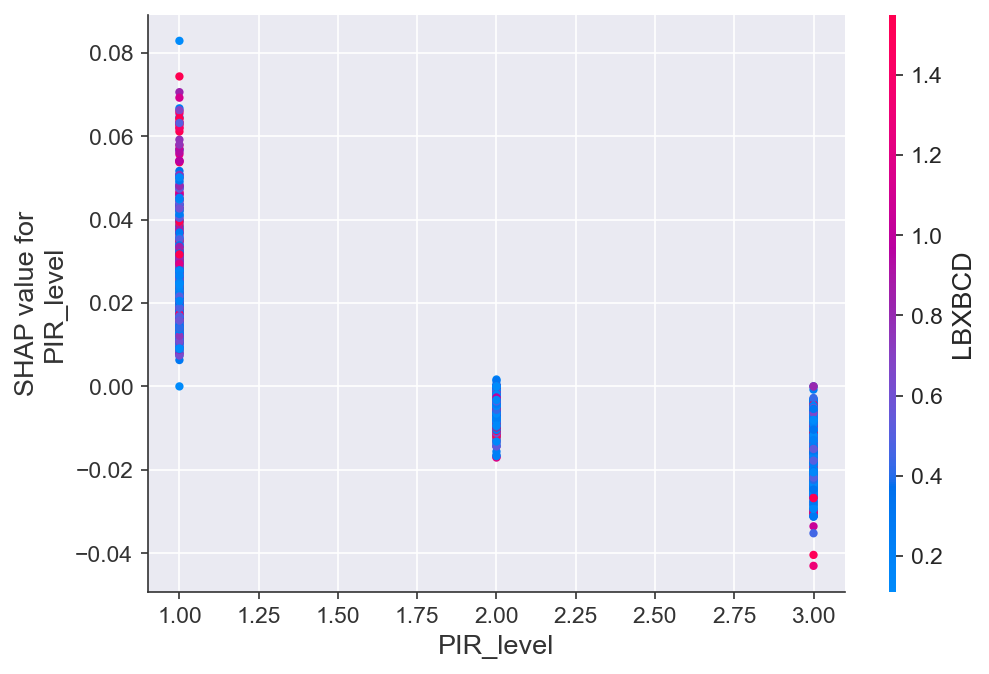

Supplement: Supplementary file 1 [file Data_Sheet_1.zip › dependence/PIR_level.png]

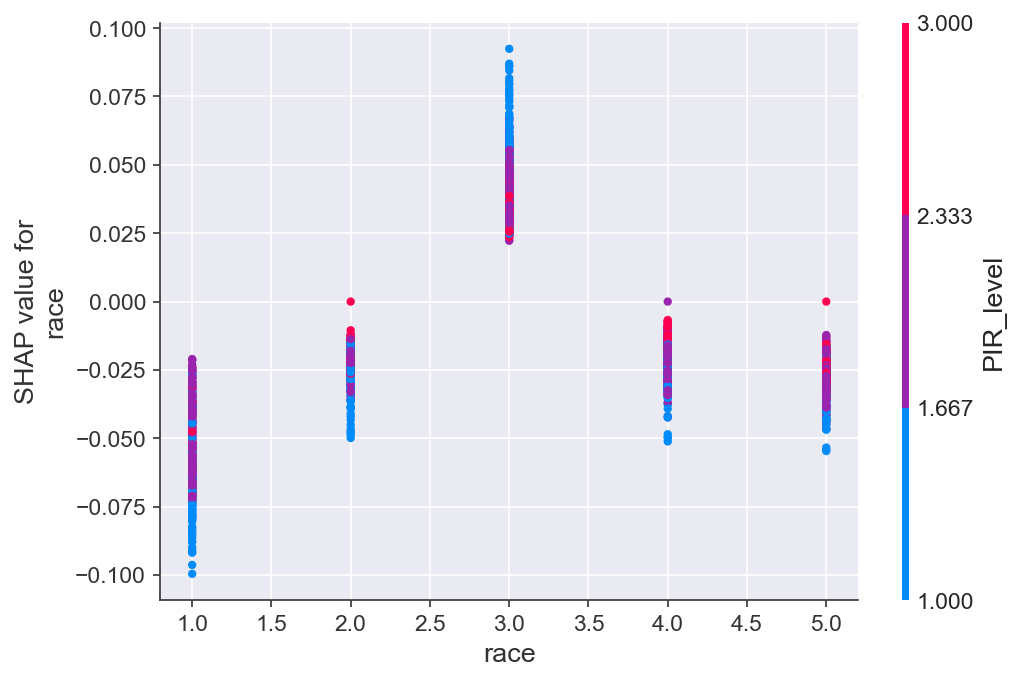

Supplement: Supplementary file 1 [file Data_Sheet_1.zip › dependence/race.png]

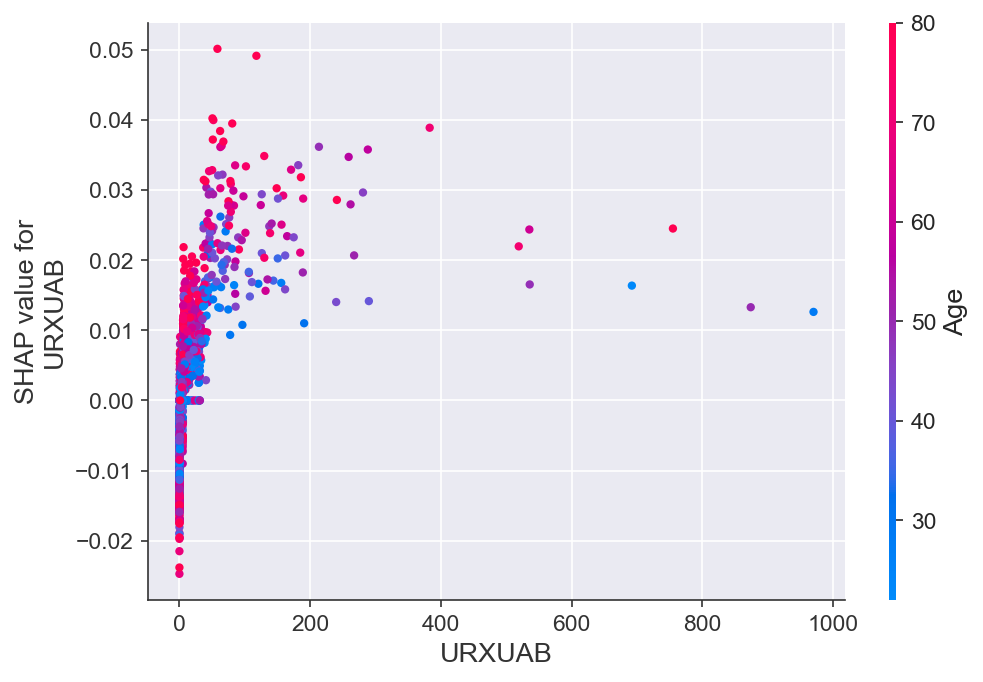

Supplement: Supplementary file 1 [file Data_Sheet_1.zip › dependence/URXUAB.png]

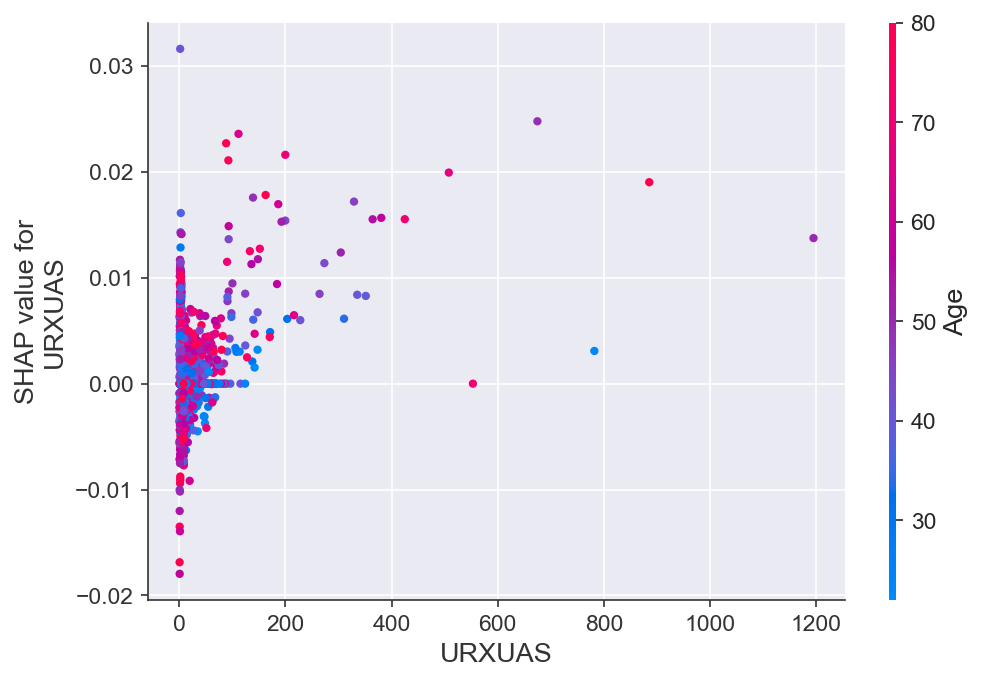

Supplement: Supplementary file 1 [file Data_Sheet_1.zip › dependence/URXUAS.png]

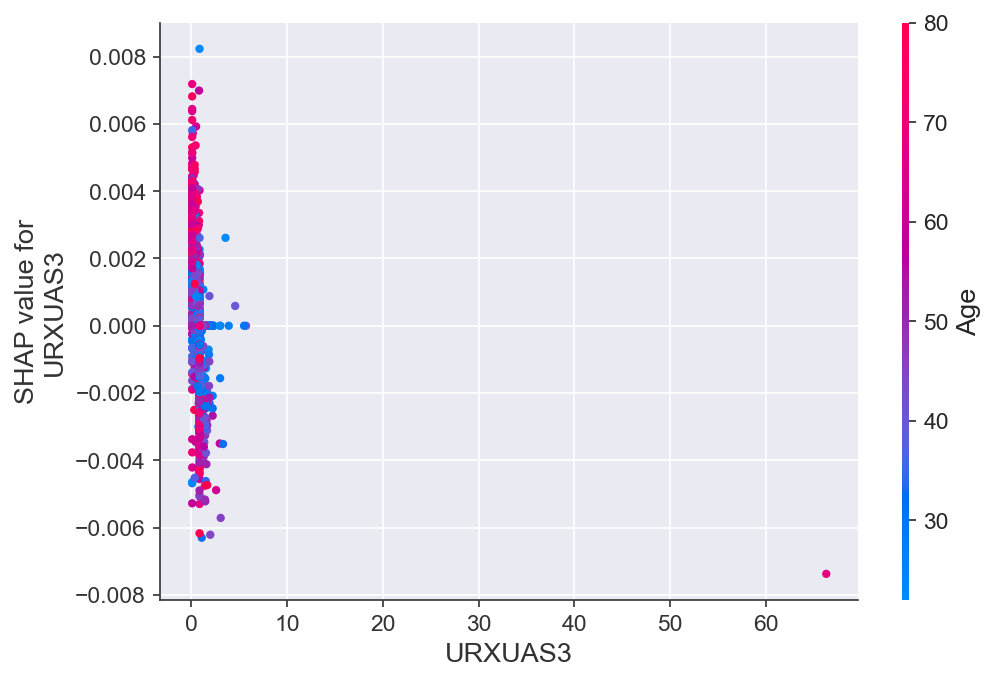

Supplement: Supplementary file 1 [file Data_Sheet_1.zip › dependence/URXUAS3.png]

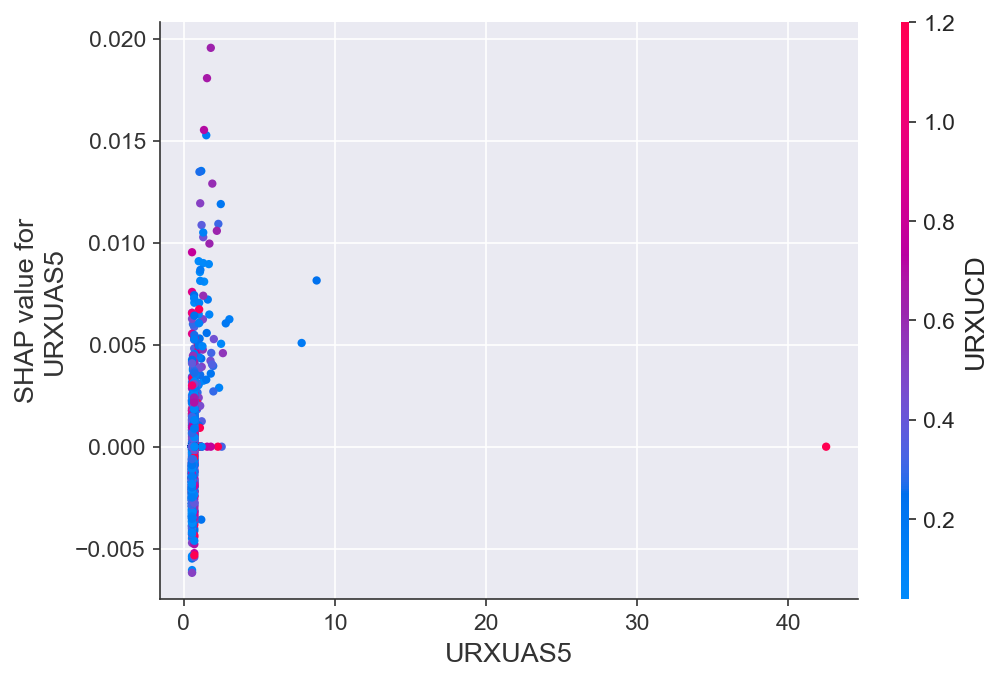

Supplement: Supplementary file 1 [file Data_Sheet_1.zip › dependence/URXUAS5.png]

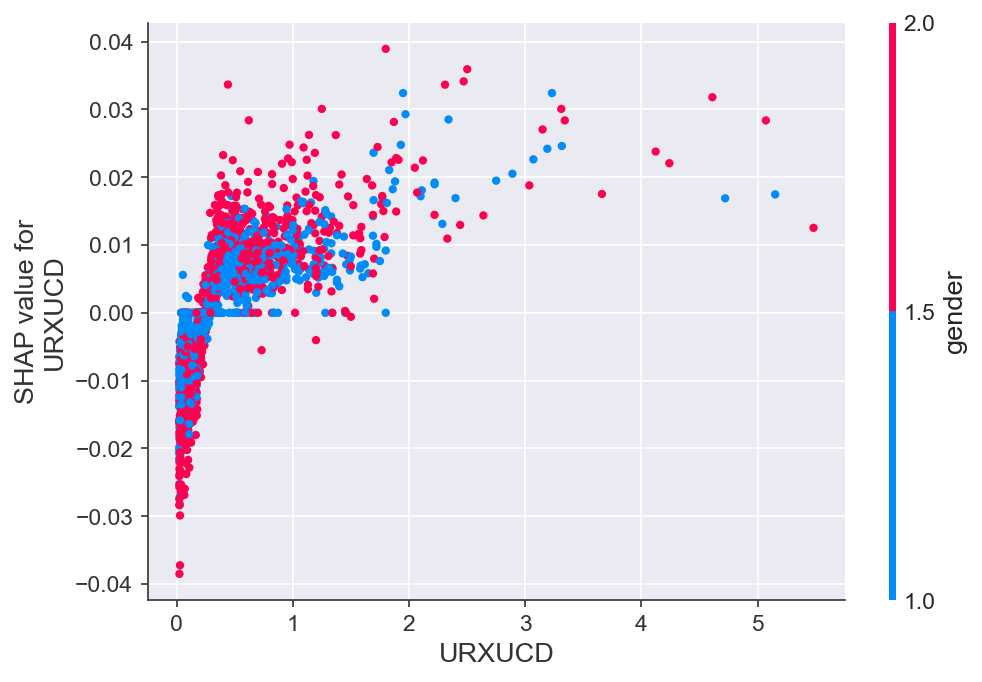

Supplement: Supplementary file 1 [file Data_Sheet_1.zip › dependence/URXUCD.png]

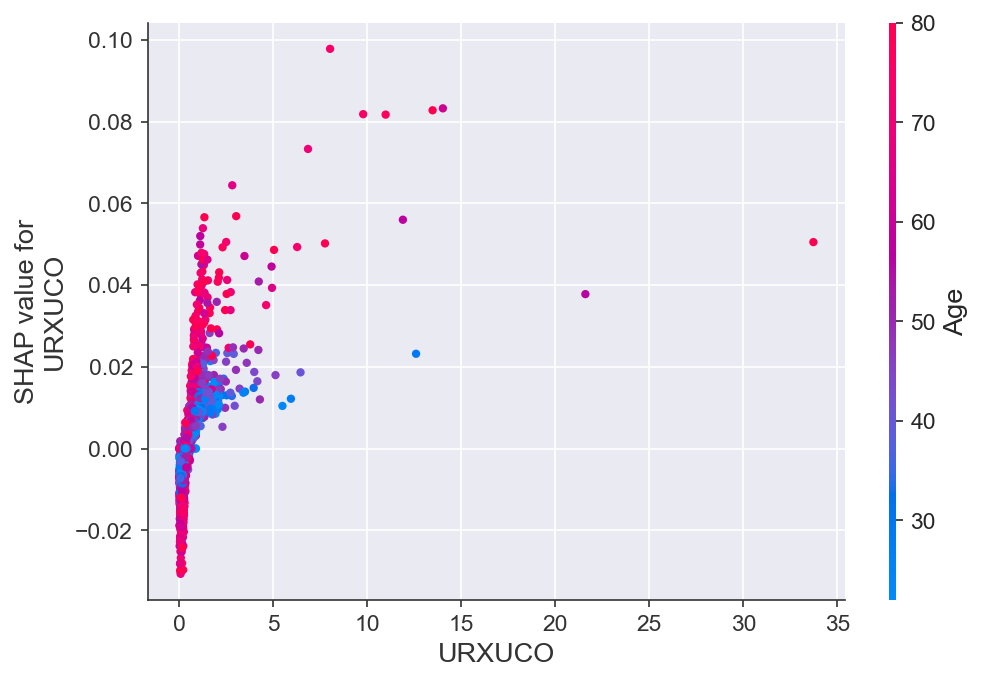

Supplement: Supplementary file 1 [file Data_Sheet_1.zip › dependence/URXUCO.png]

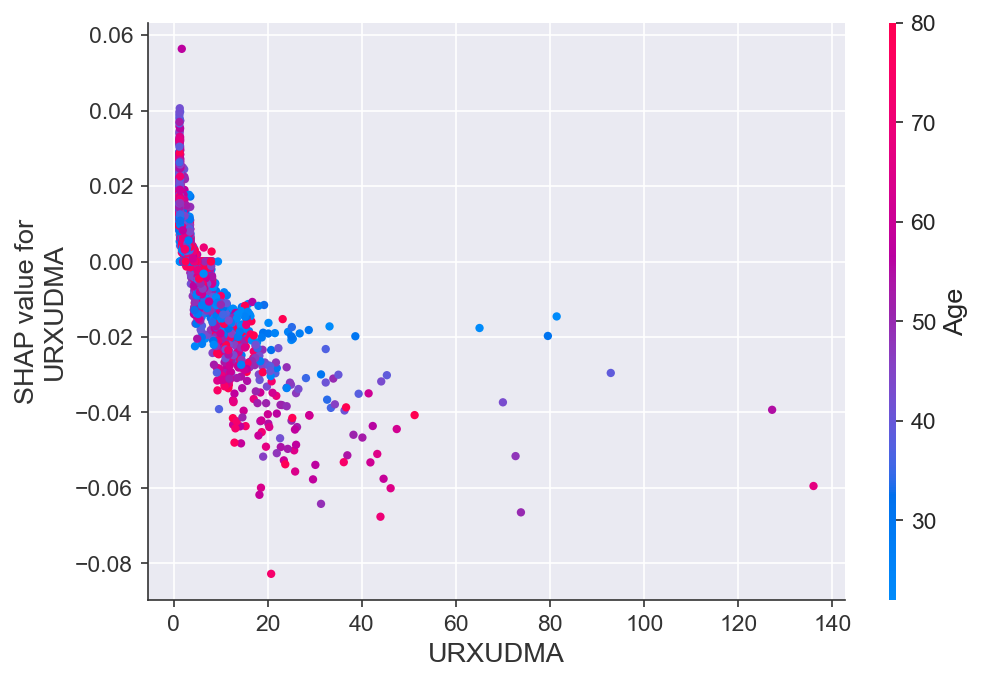

Supplement: Supplementary file 1 [file Data_Sheet_1.zip › dependence/URXUDMA.png]

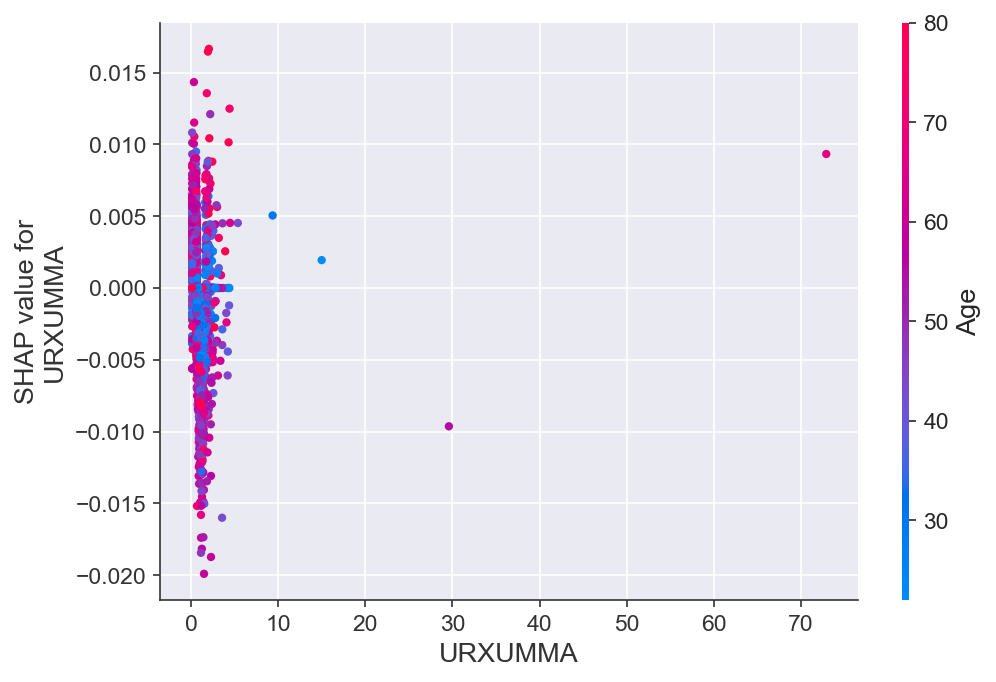

Supplement: Supplementary file 1 [file Data_Sheet_1.zip › dependence/URXUMMA.png]

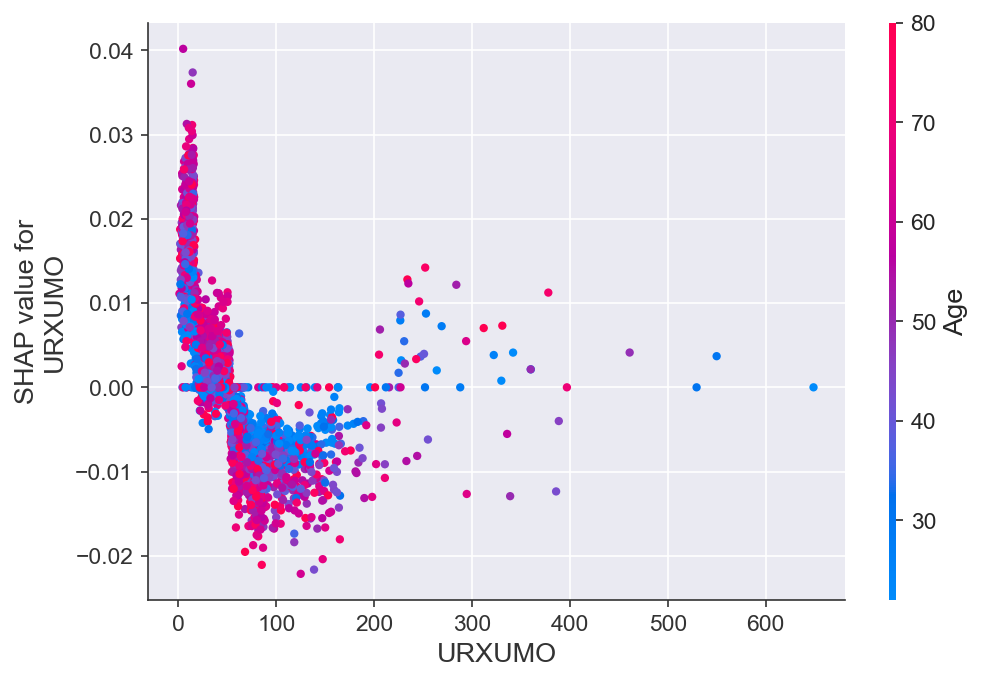

Supplement: Supplementary file 1 [file Data_Sheet_1.zip › dependence/URXUMO.png]

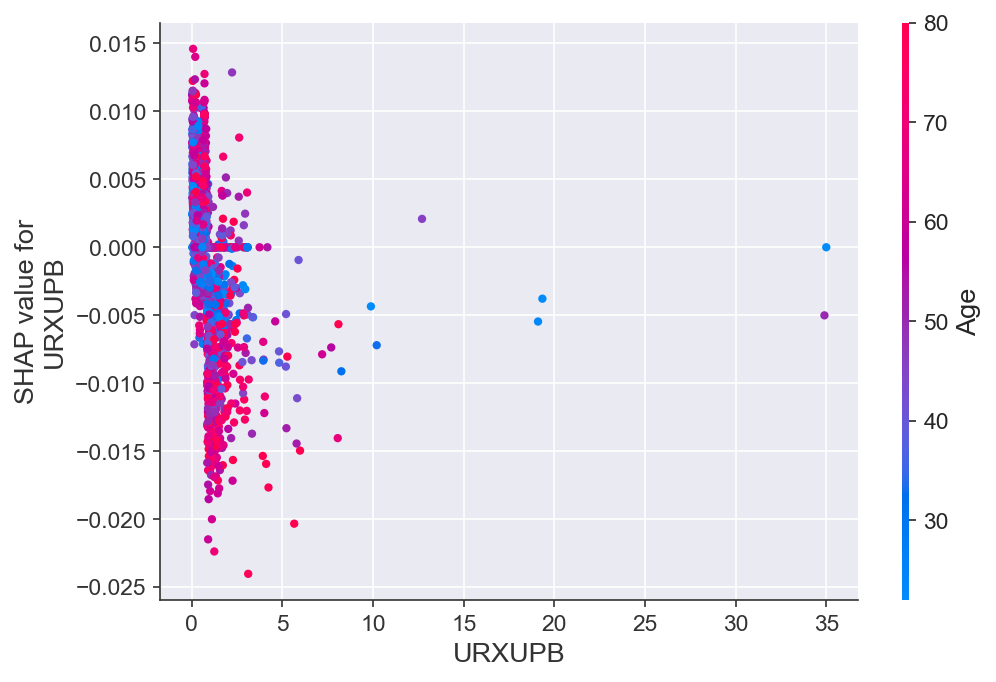

Supplement: Supplementary file 1 [file Data_Sheet_1.zip › dependence/URXUPB.png]

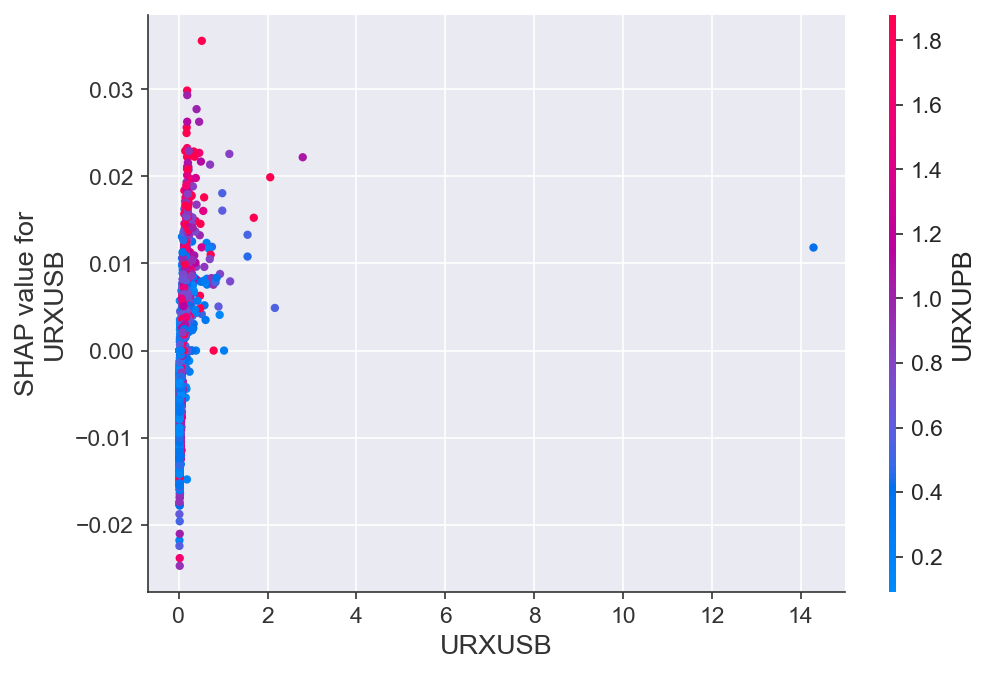

Supplement: Supplementary file 1 [file Data_Sheet_1.zip › dependence/URXUSB.png]

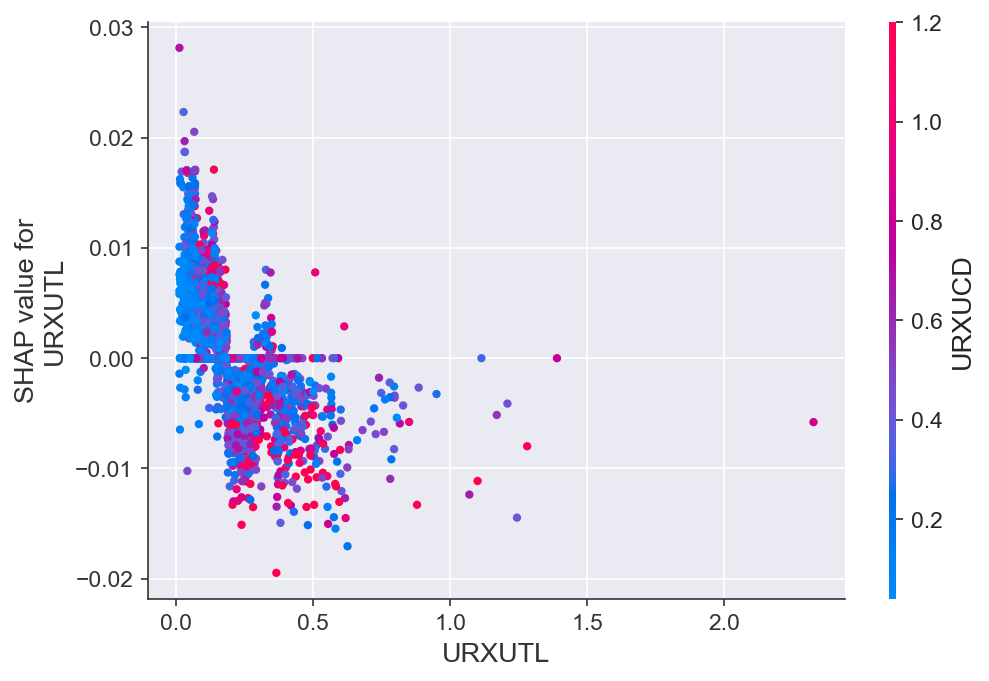

Supplement: Supplementary file 1 [file Data_Sheet_1.zip › dependence/URXUTL.png]

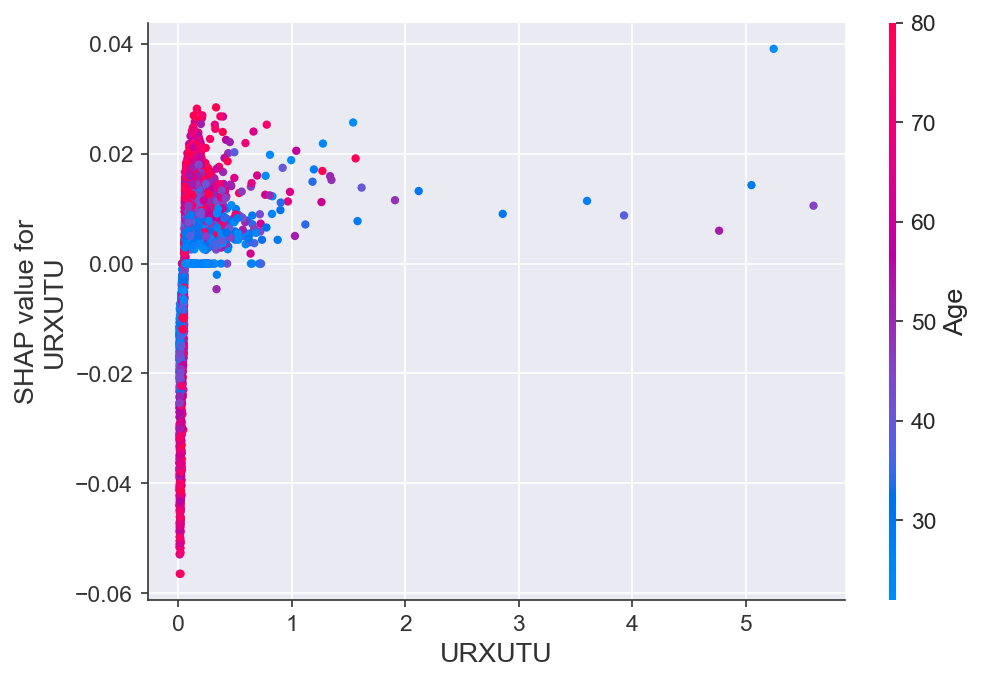

Supplement: Supplementary file 1 [file Data_Sheet_1.zip › dependence/URXUTU.png]

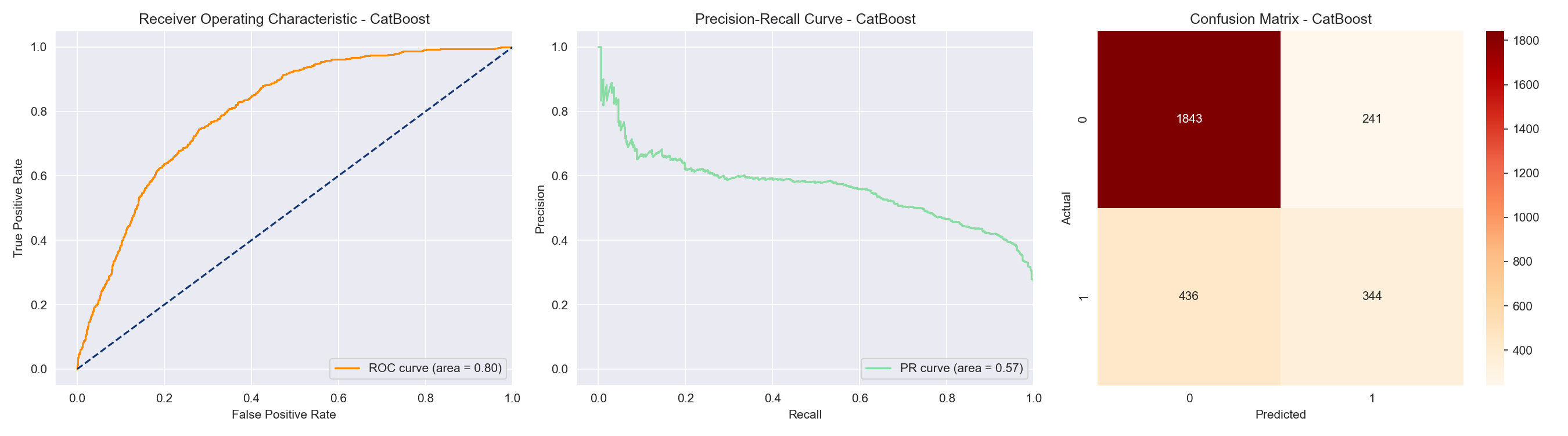

Supplement: Supplementary file 1 [file Data_Sheet_1.zip › Figure1-13 (arthritis_status_13Model)/ROC_PR_CM_CatBoost.png]

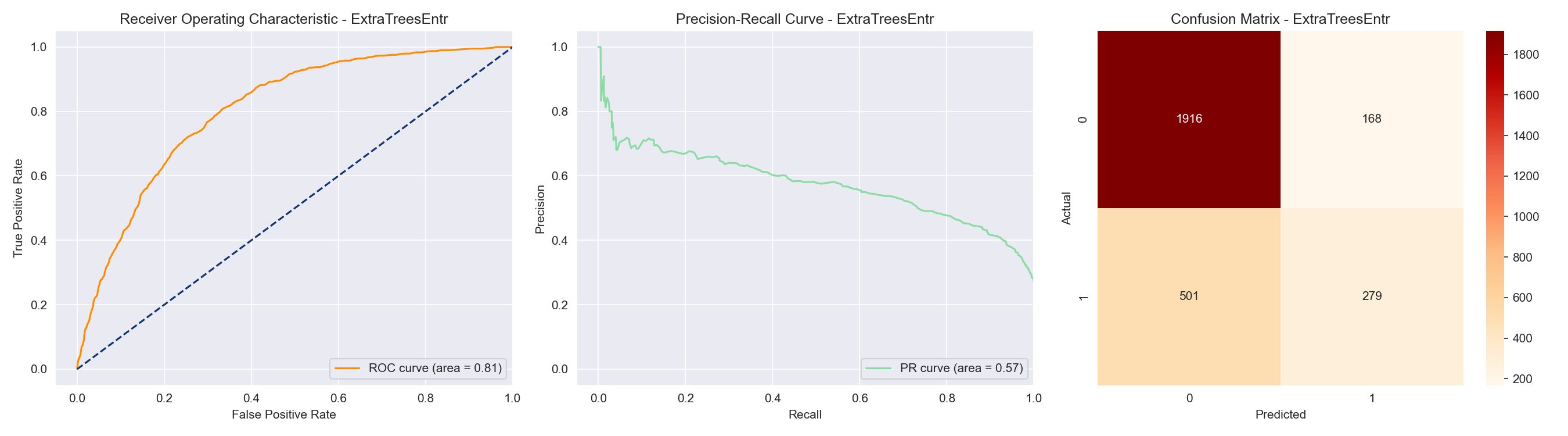

Supplement: Supplementary file 1 [file Data_Sheet_1.zip › Figure1-13 (arthritis_status_13Model)/ROC_PR_CM_ExtraTreesEntr.png]

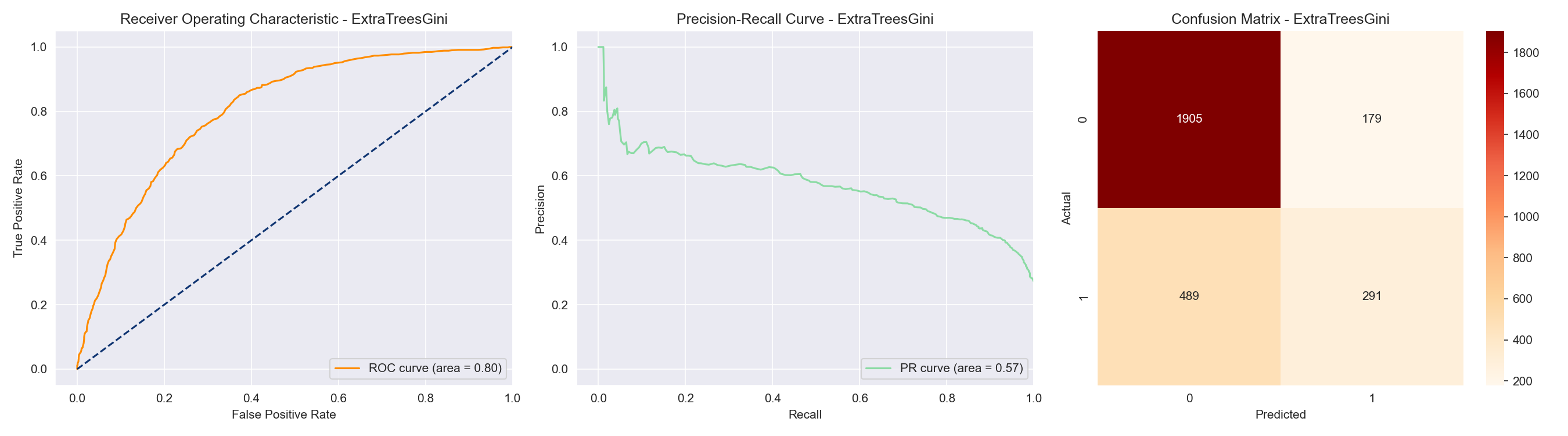

Supplement: Supplementary file 1 [file Data_Sheet_1.zip › Figure1-13 (arthritis_status_13Model)/ROC_PR_CM_ExtraTreesGini.png]

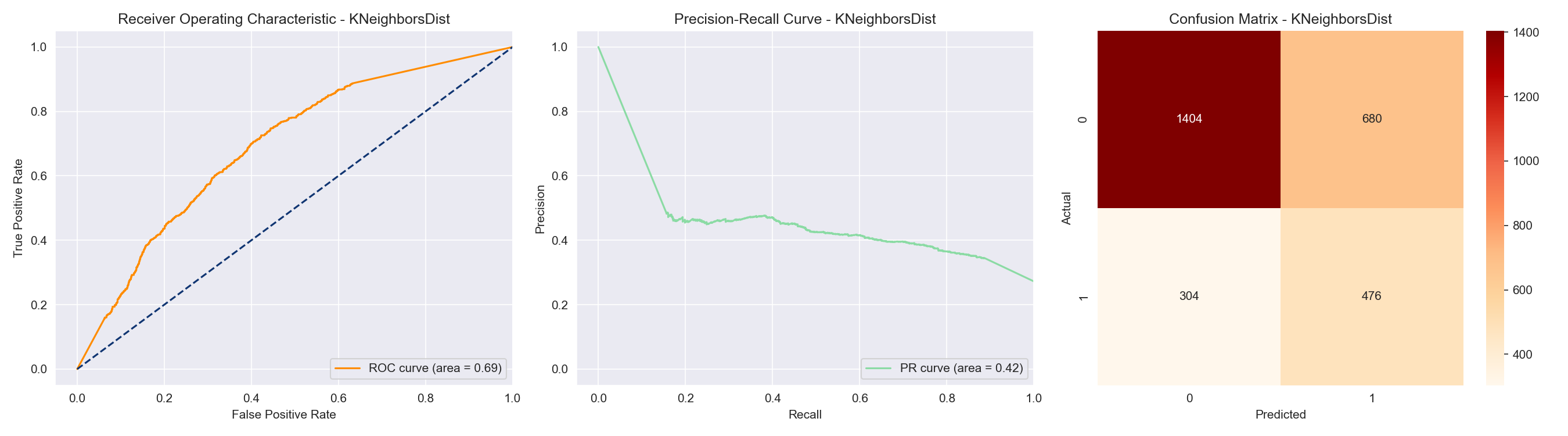

Supplement: Supplementary file 1 [file Data_Sheet_1.zip › Figure1-13 (arthritis_status_13Model)/ROC_PR_CM_KNeighborsDist.png]

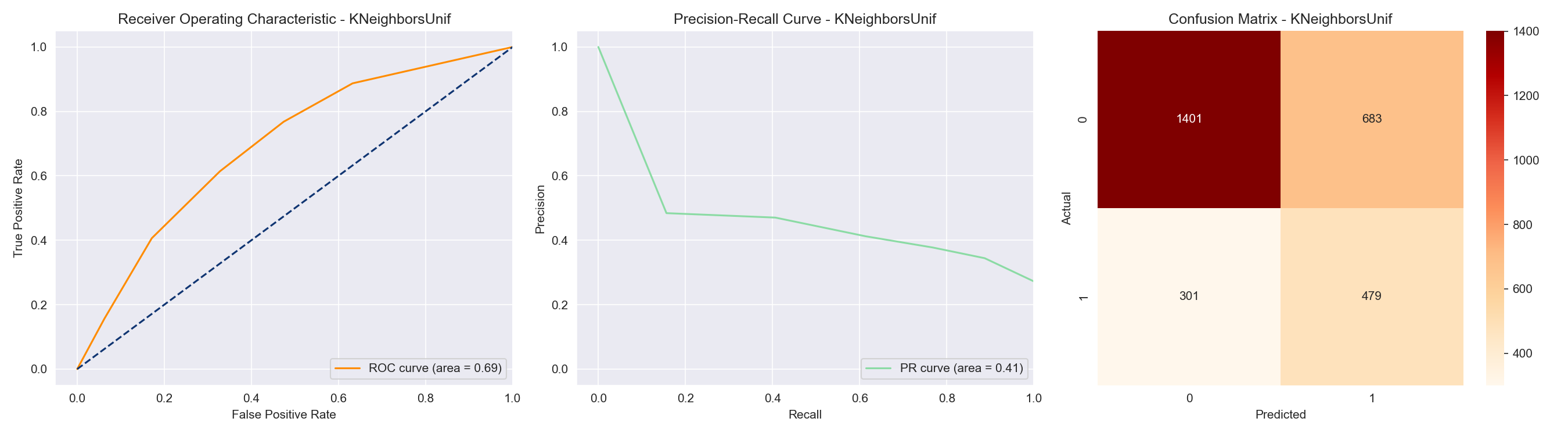

Supplement: Supplementary file 1 [file Data_Sheet_1.zip › Figure1-13 (arthritis_status_13Model)/ROC_PR_CM_KNeighborsUnif.png]

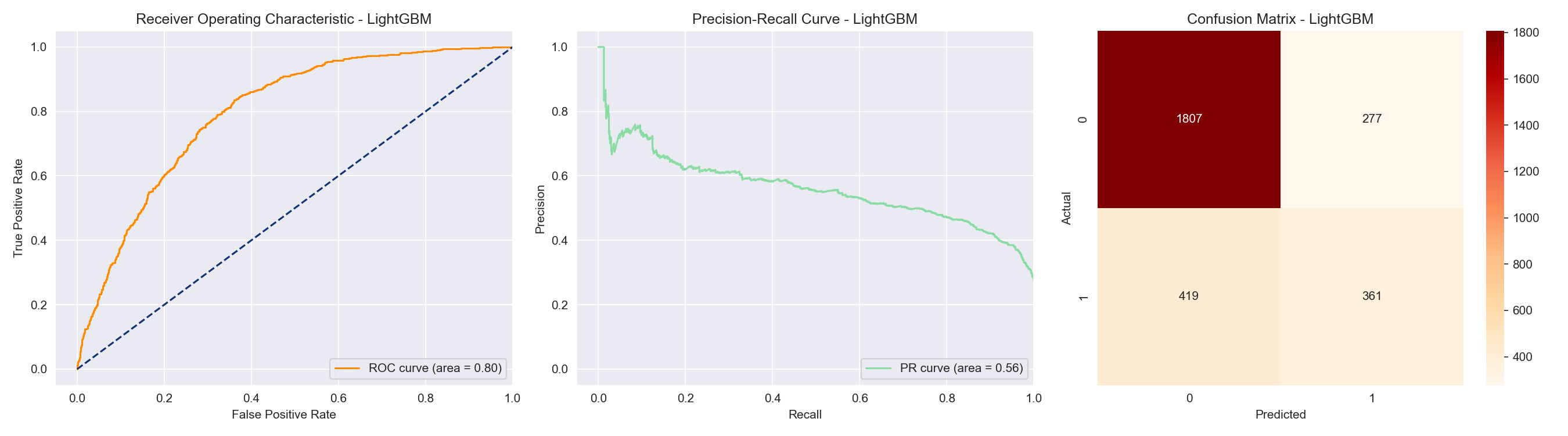

Supplement: Supplementary file 1 [file Data_Sheet_1.zip › Figure1-13 (arthritis_status_13Model)/ROC_PR_CM_LightGBM.png]

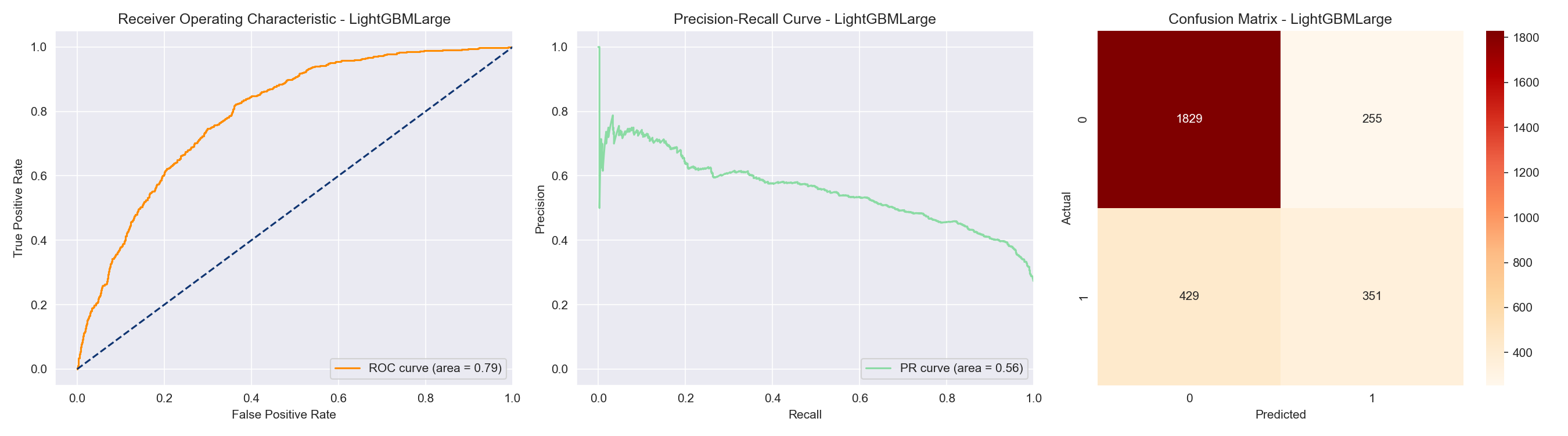

Supplement: Supplementary file 1 [file Data_Sheet_1.zip › Figure1-13 (arthritis_status_13Model)/ROC_PR_CM_LightGBMLarge.png]

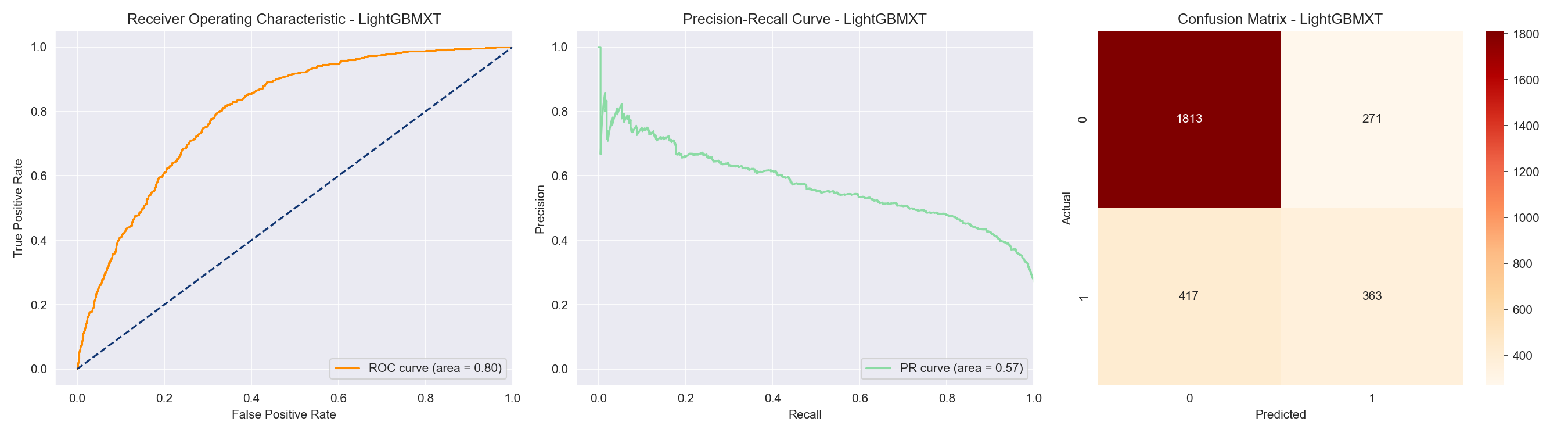

Supplement: Supplementary file 1 [file Data_Sheet_1.zip › Figure1-13 (arthritis_status_13Model)/ROC_PR_CM_LightGBMXT.png]

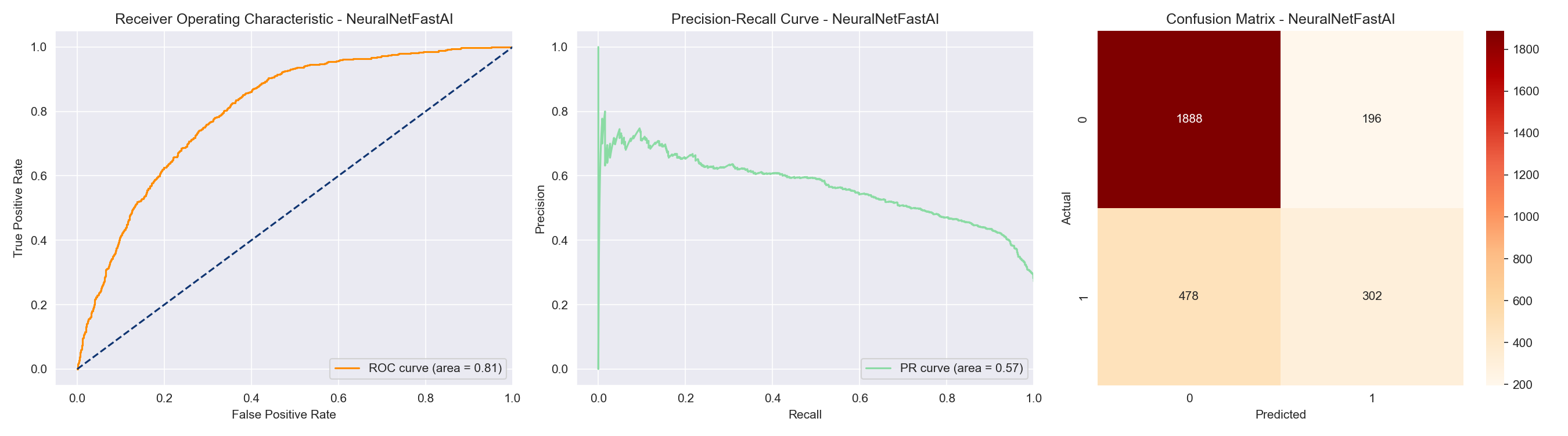

Supplement: Supplementary file 1 [file Data_Sheet_1.zip › Figure1-13 (arthritis_status_13Model)/ROC_PR_CM_NeuralNetFastAI.png]

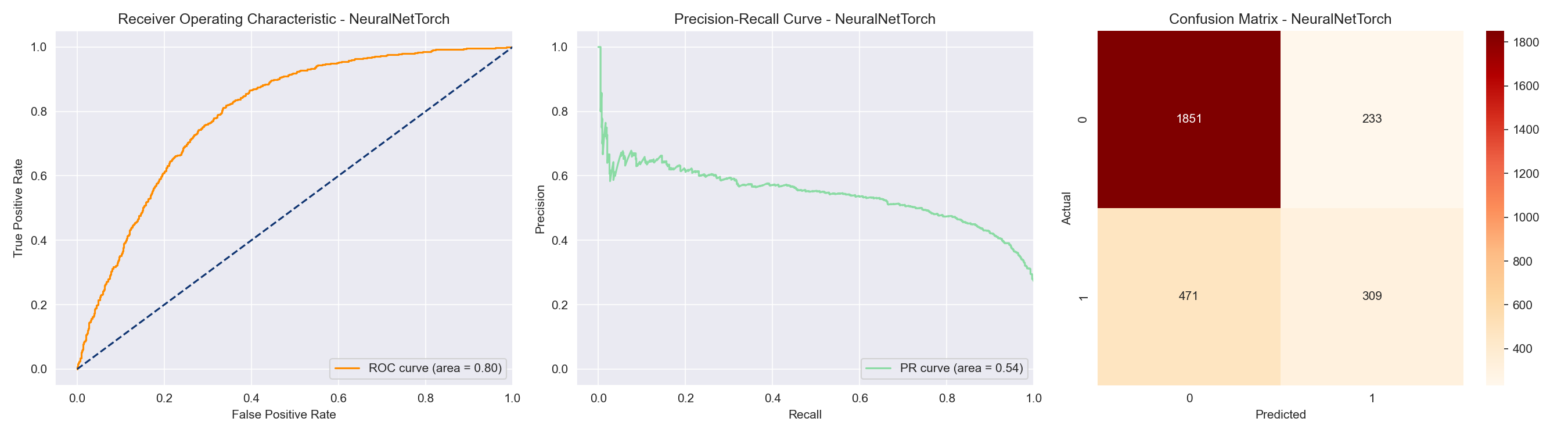

Supplement: Supplementary file 1 [file Data_Sheet_1.zip › Figure1-13 (arthritis_status_13Model)/ROC_PR_CM_NeuralNetTorch.png]

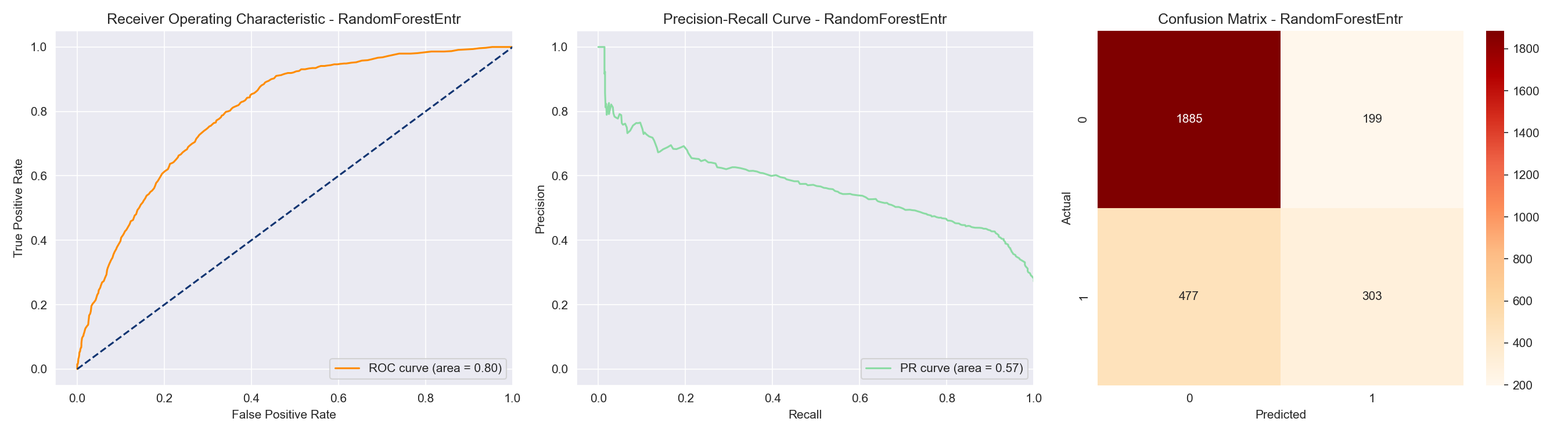

Supplement: Supplementary file 1 [file Data_Sheet_1.zip › Figure1-13 (arthritis_status_13Model)/ROC_PR_CM_RandomForestEntr.png]

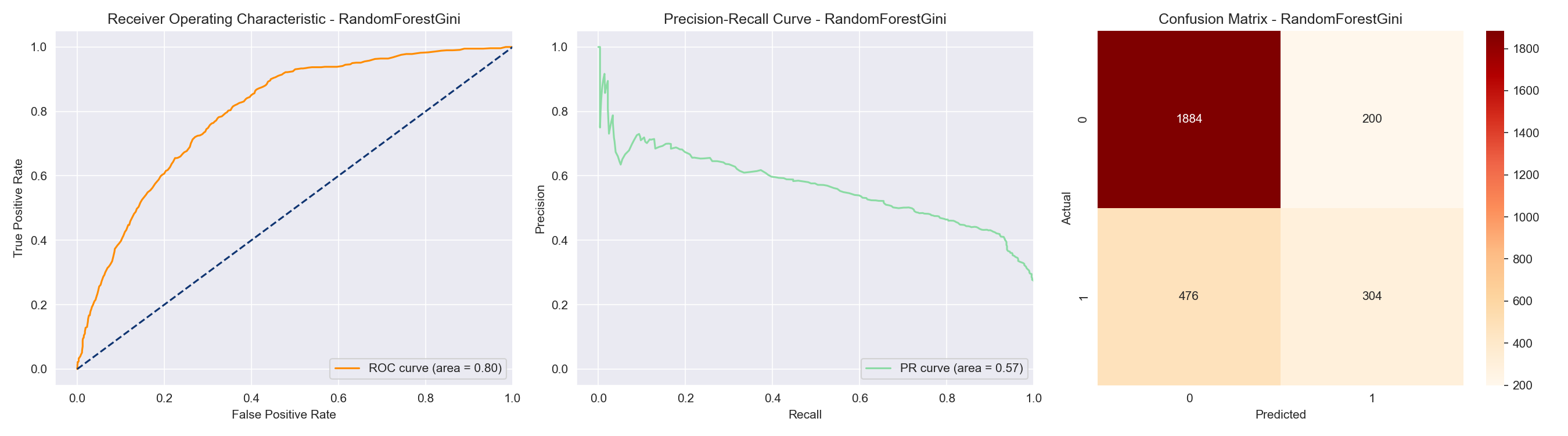

Supplement: Supplementary file 1 [file Data_Sheet_1.zip › Figure1-13 (arthritis_status_13Model)/ROC_PR_CM_RandomForestGini.png]

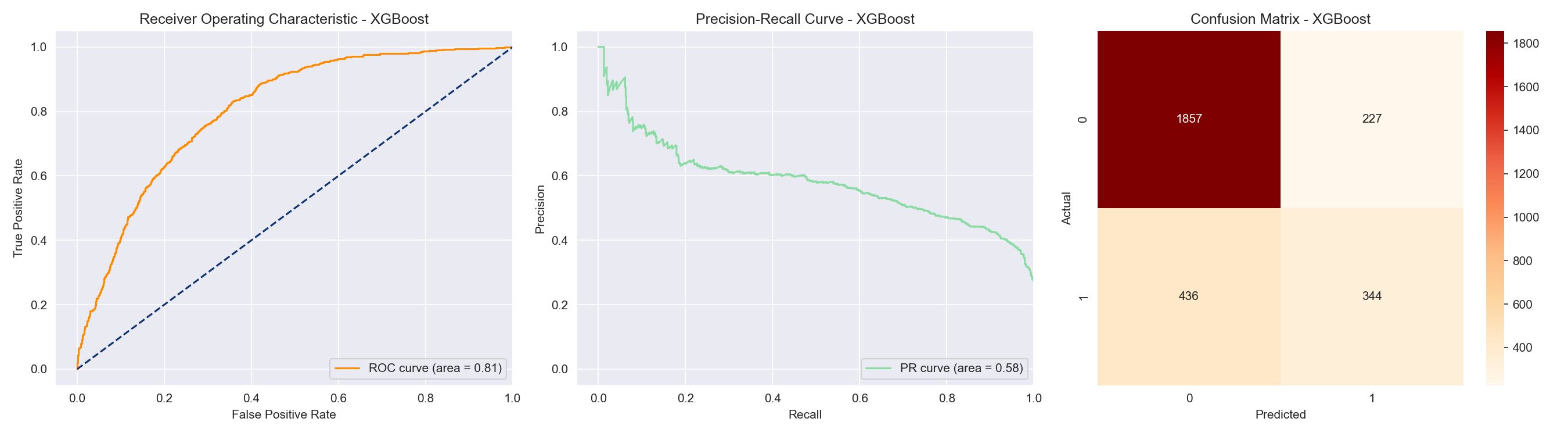

Supplement: Supplementary file 1 [file Data_Sheet_1.zip › Figure1-13 (arthritis_status_13Model)/ROC_PR_CM_XGBoost.png]

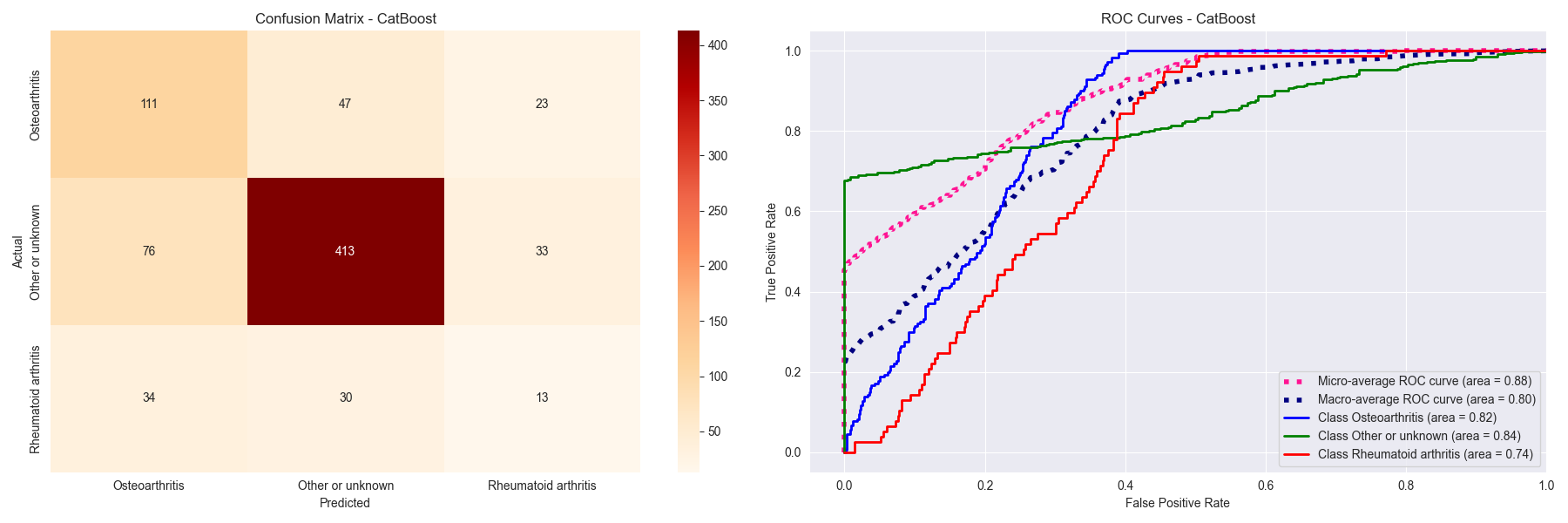

Supplement: Supplementary file 1 [file Data_Sheet_1.zip › Figure14-26 (OARA_13Model)/CM_ROC_CatBoost.png]

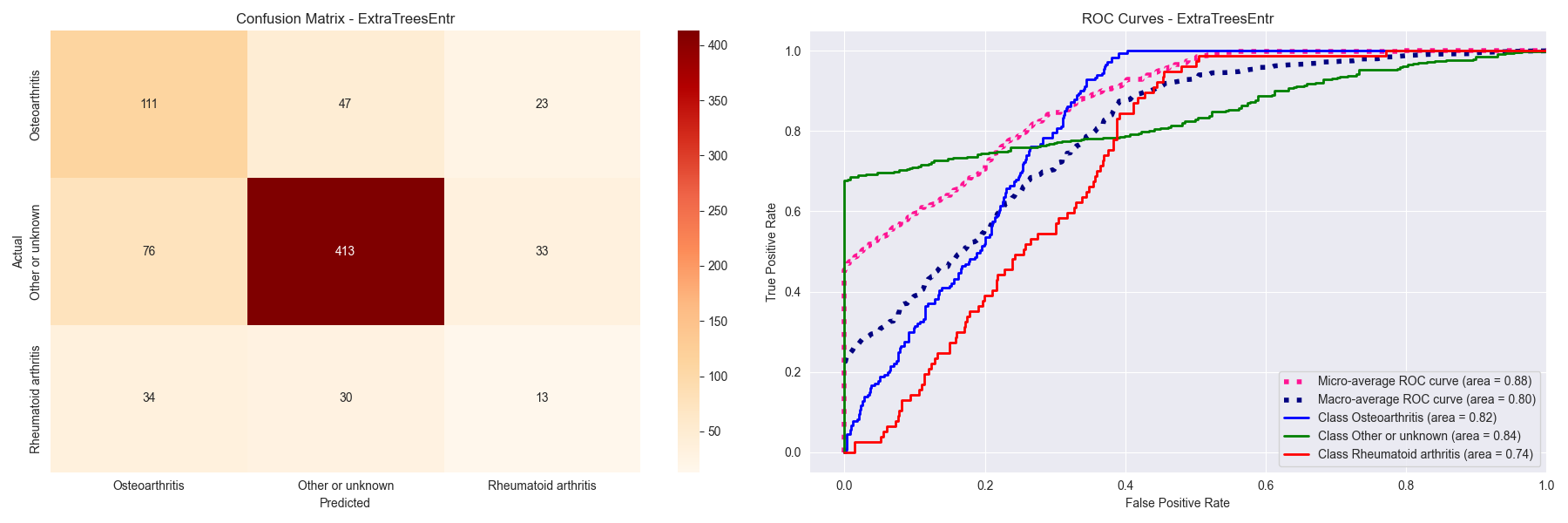

Supplement: Supplementary file 1 [file Data_Sheet_1.zip › Figure14-26 (OARA_13Model)/CM_ROC_ExtraTreesEntr.png]

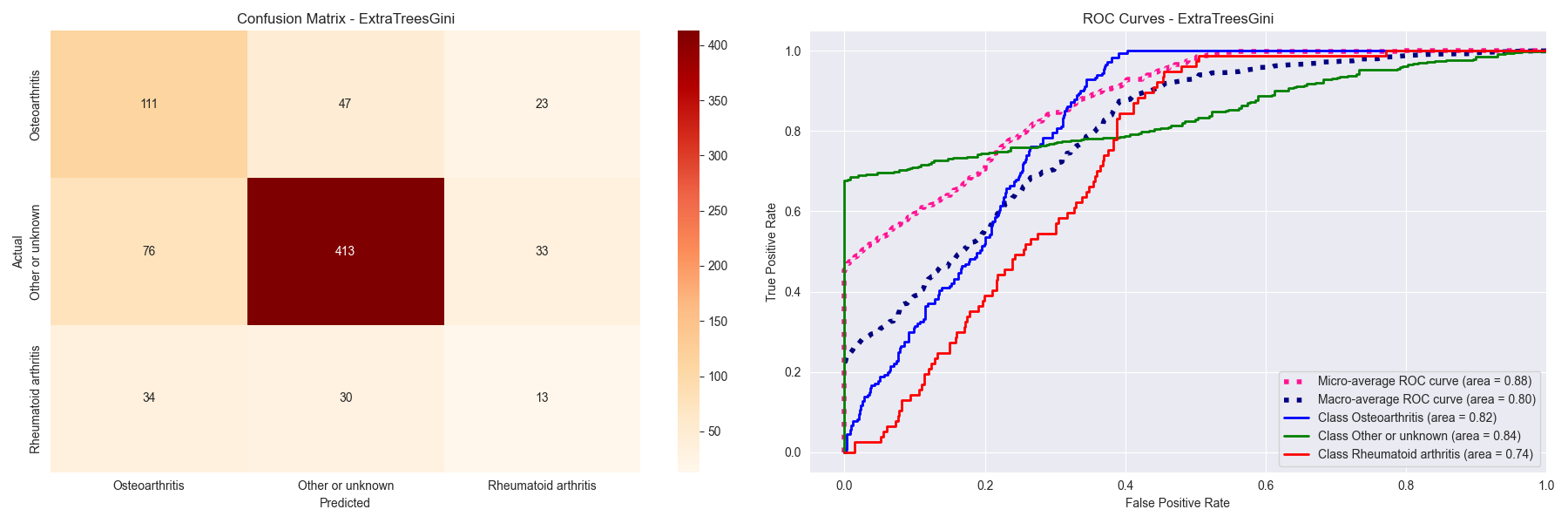

Supplement: Supplementary file 1 [file Data_Sheet_1.zip › Figure14-26 (OARA_13Model)/CM_ROC_ExtraTreesGini.png]

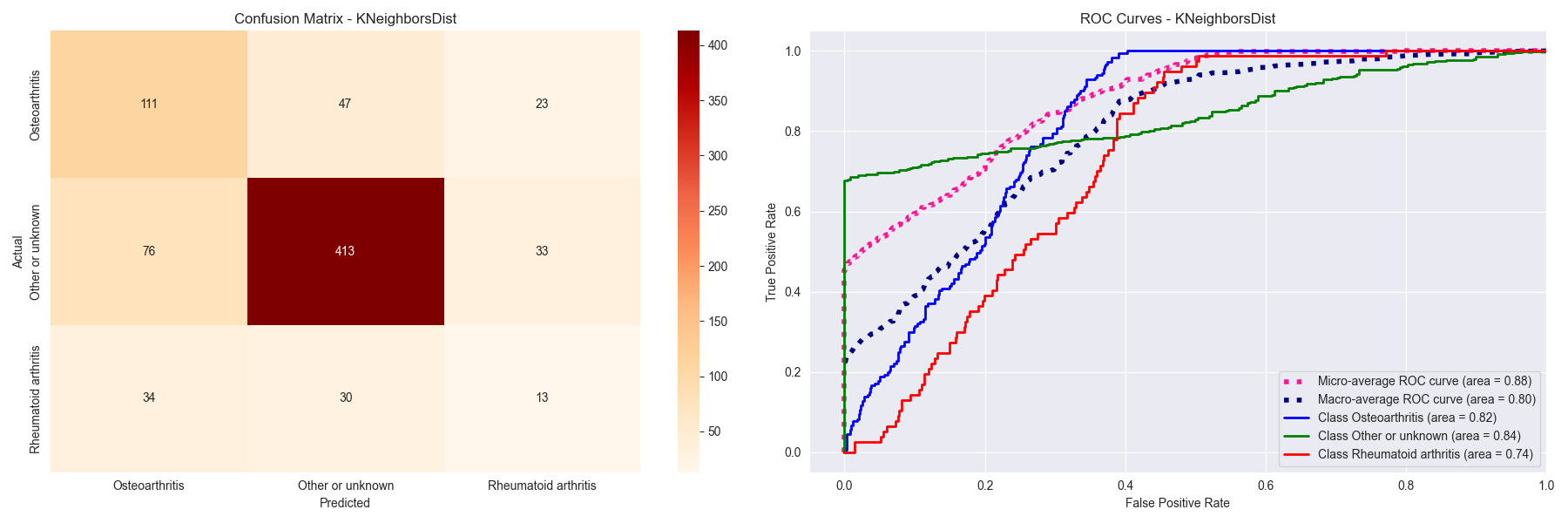

Supplement: Supplementary file 1 [file Data_Sheet_1.zip › Figure14-26 (OARA_13Model)/CM_ROC_KNeighborsDist.png]

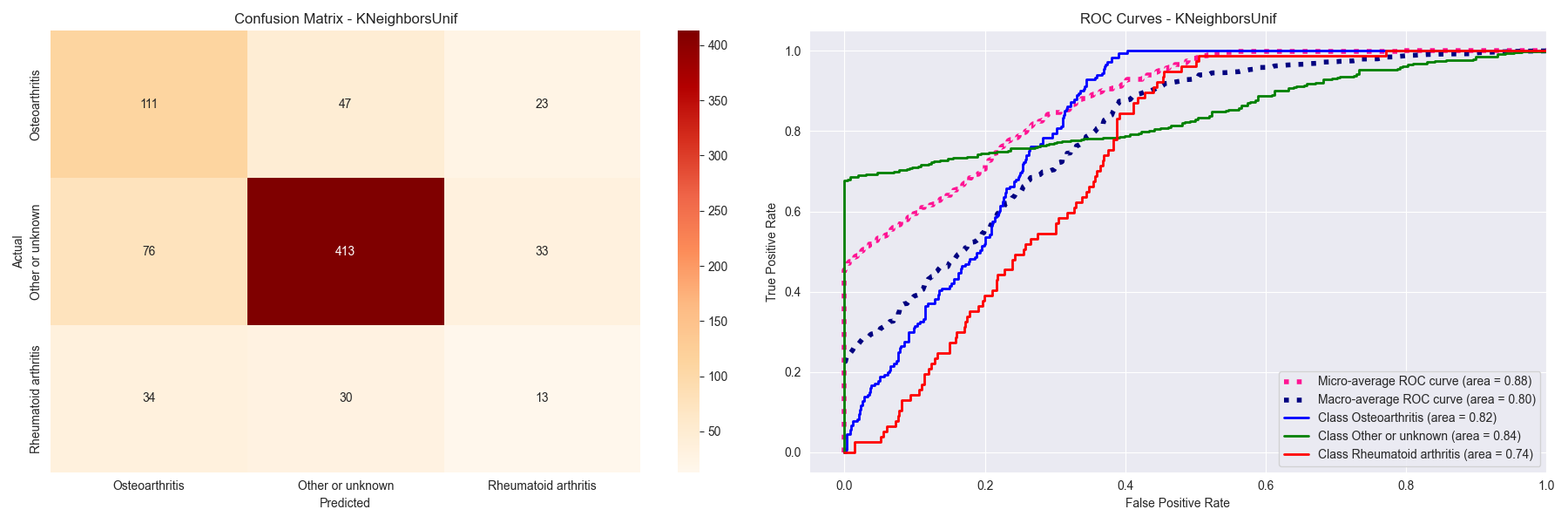

Supplement: Supplementary file 1 [file Data_Sheet_1.zip › Figure14-26 (OARA_13Model)/CM_ROC_KNeighborsUnif.png]

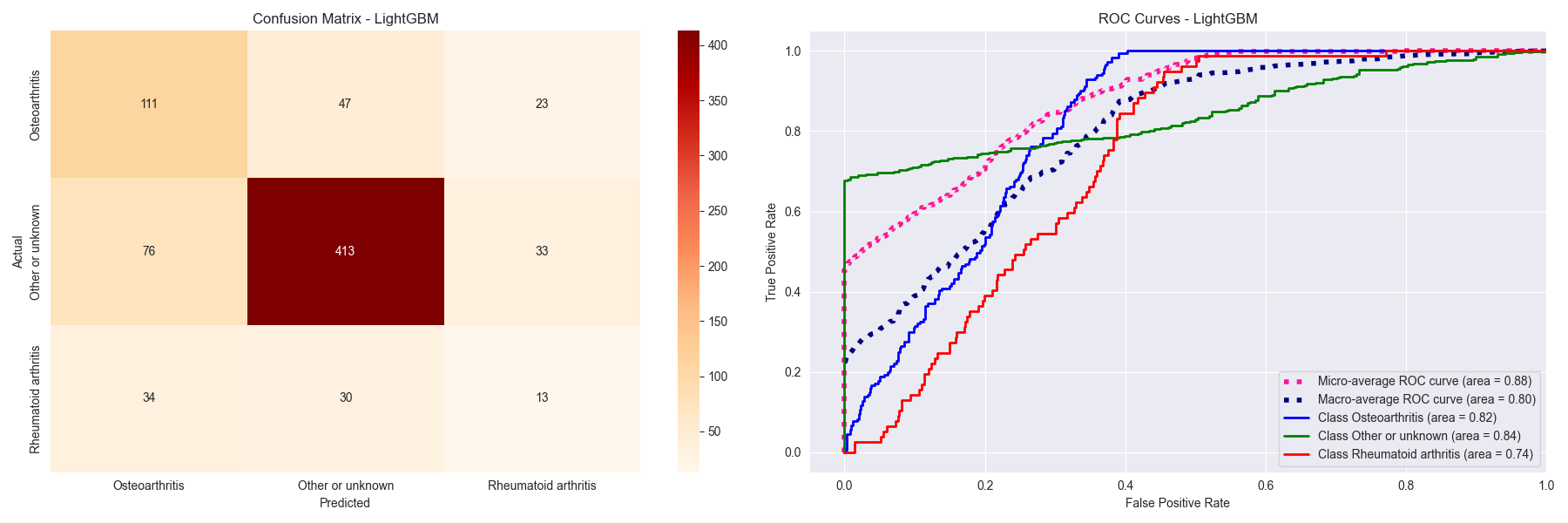

Supplement: Supplementary file 1 [file Data_Sheet_1.zip › Figure14-26 (OARA_13Model)/CM_ROC_LightGBM.png]

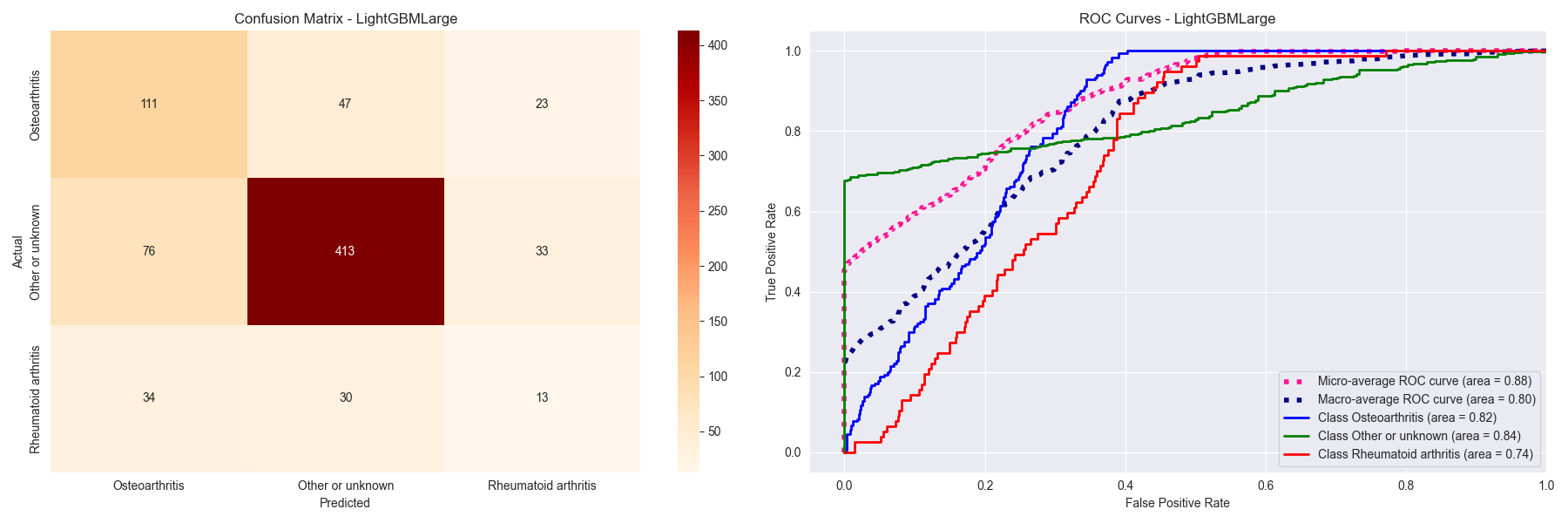

Supplement: Supplementary file 1 [file Data_Sheet_1.zip › Figure14-26 (OARA_13Model)/CM_ROC_LightGBMLarge.png]

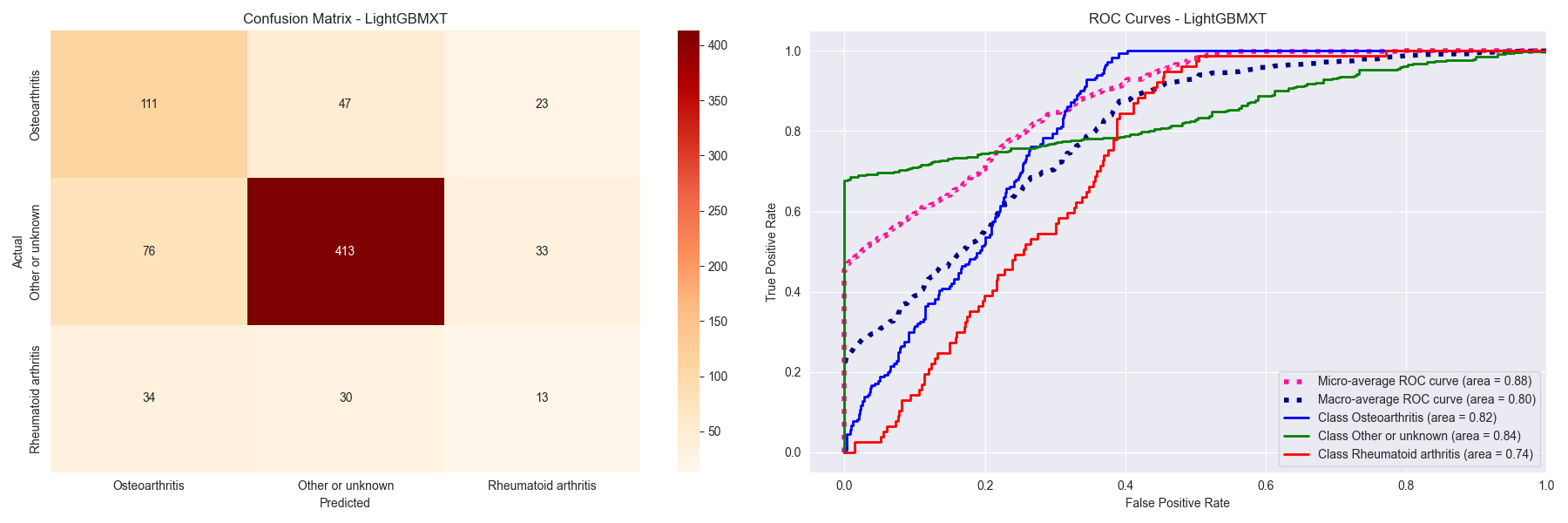

Supplement: Supplementary file 1 [file Data_Sheet_1.zip › Figure14-26 (OARA_13Model)/CM_ROC_LightGBMXT.png]

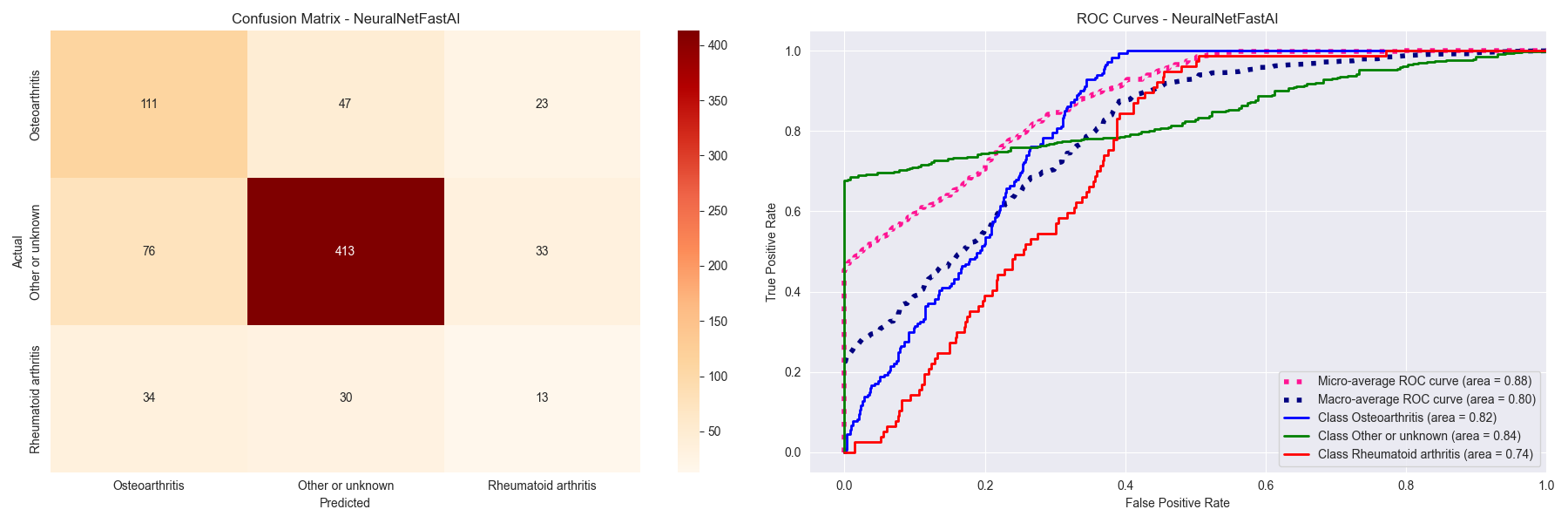

Supplement: Supplementary file 1 [file Data_Sheet_1.zip › Figure14-26 (OARA_13Model)/CM_ROC_NeuralNetFastAI.png]

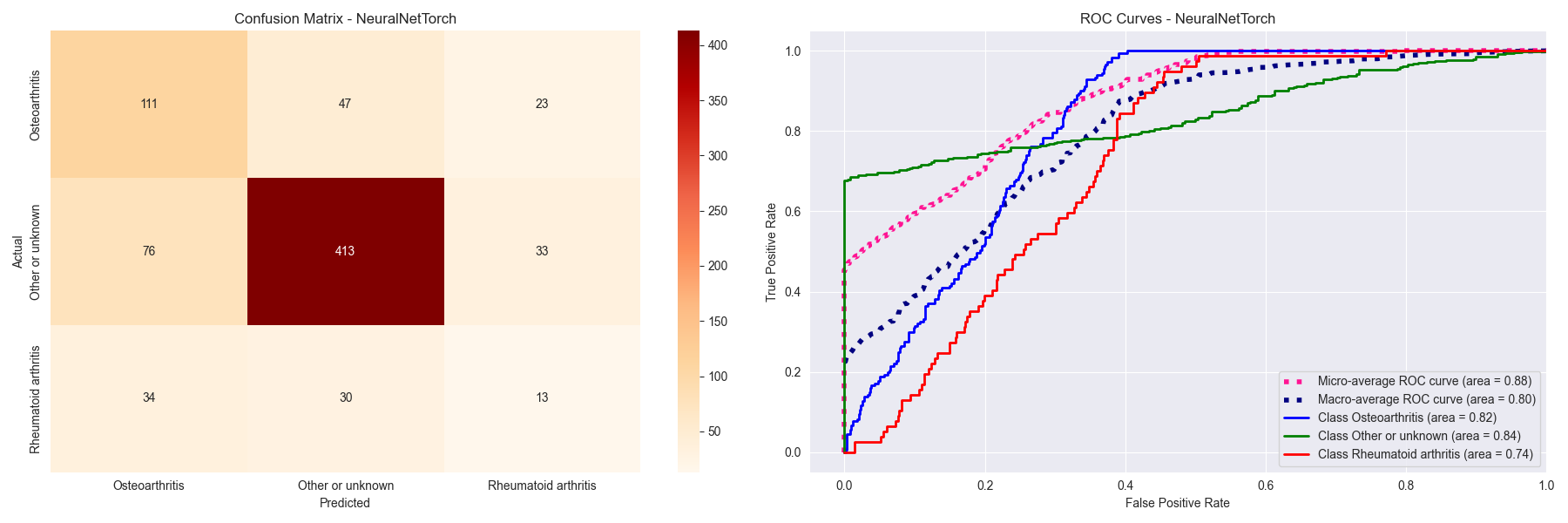

Supplement: Supplementary file 1 [file Data_Sheet_1.zip › Figure14-26 (OARA_13Model)/CM_ROC_NeuralNetTorch.png]

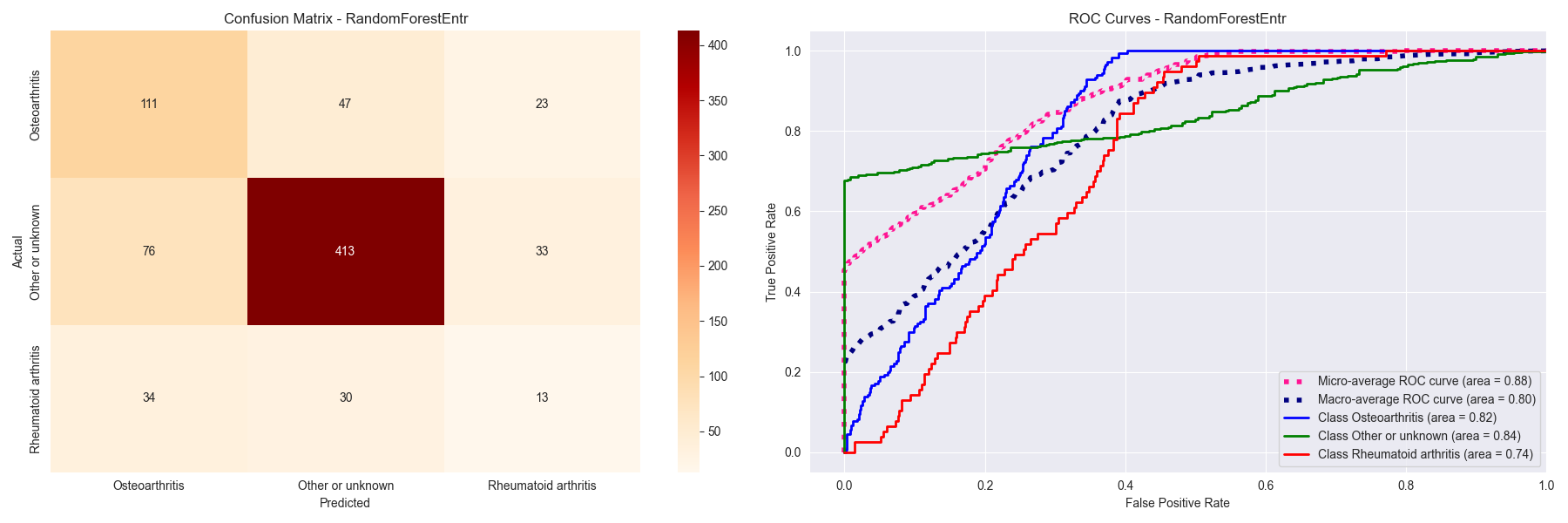

Supplement: Supplementary file 1 [file Data_Sheet_1.zip › Figure14-26 (OARA_13Model)/CM_ROC_RandomForestEntr.png]

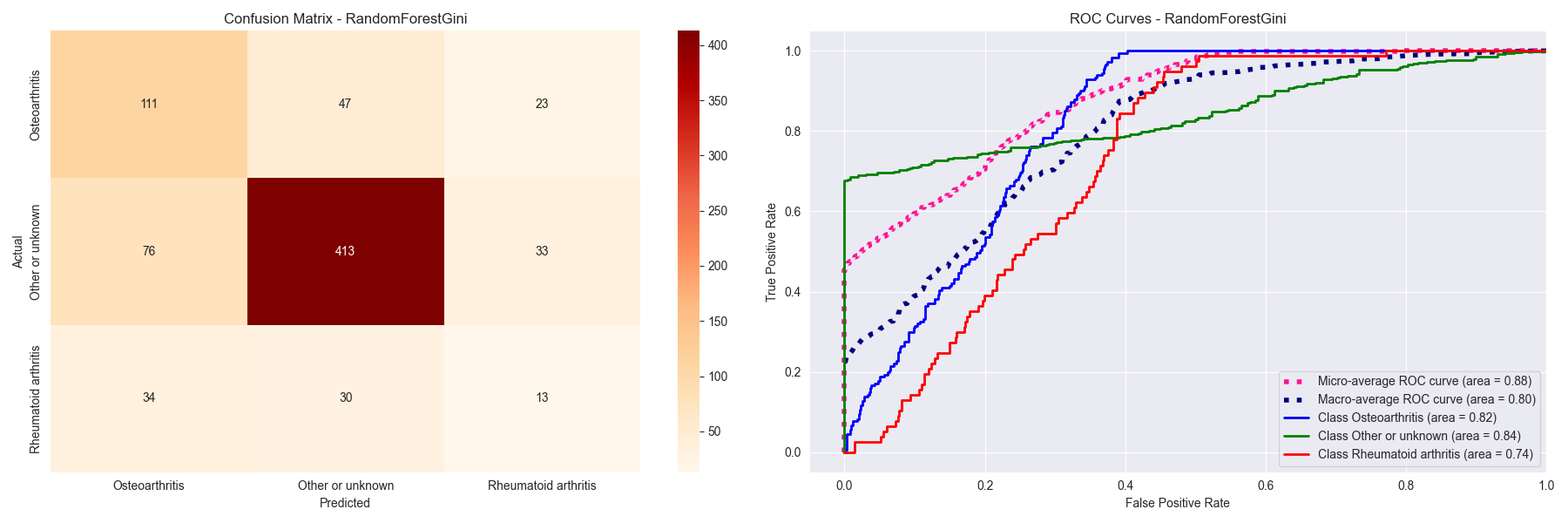

Supplement: Supplementary file 1 [file Data_Sheet_1.zip › Figure14-26 (OARA_13Model)/CM_ROC_RandomForestGini.png]

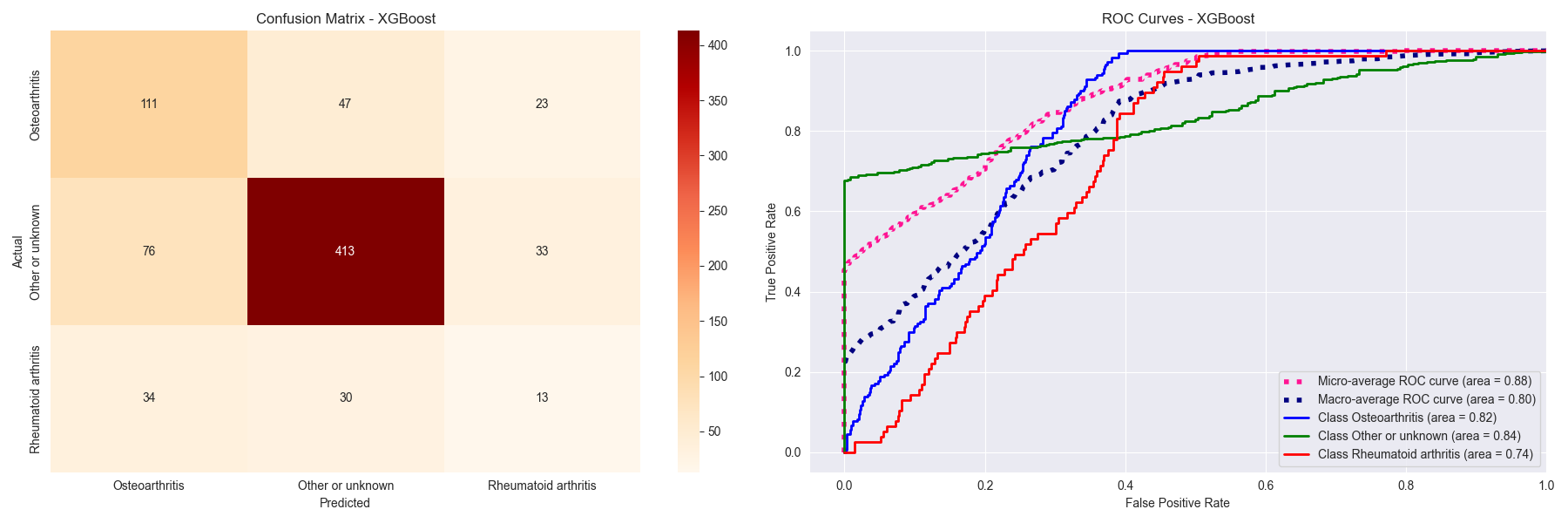

Supplement: Supplementary file 1 [file Data_Sheet_1.zip › Figure14-26 (OARA_13Model)/CM_ROC_XGBoost.png]
